# Adaptation of the master antioxidant response connects metabolism, lifespan and feather development pathways in birds

**DOI:** 10.1038/s41467-020-16129-4

**Published:** 2020-05-18

**Authors:** Gianni M. Castiglione, Zhenhua Xu, Lingli Zhou, Elia J. Duh

**Affiliations:** Department of Ophthalmology, Johns Hopkins University School of Medicine, 400 N Broadway, Baltimore, MD 21287 USA

**Keywords:** Cell signalling, Behavioural ecology, Molecular evolution, Animal physiology

## Abstract

Birds (Aves) display high metabolic rates and oxygen consumption relative to mammals, increasing reactive oxygen species (ROS) formation. Although excess ROS reduces lifespan by causing extensive cellular dysfunction and damage, birds are remarkably long-lived. We address this paradox by identifying the constitutive activation of the NRF2 master antioxidant response in Neoaves (~95% of bird species), providing an adaptive mechanism capable of counterbalancing high ROS levels. We demonstrate that a KEAP1 mutation in the Neoavian ancestor disrupted the repression of NRF2 by KEAP1, leading to constitutive NRF2 activity and decreased oxidative stress in wild Neoaves tissues and cells. Our evidence suggests this ancient mutation induced a compensatory program in NRF2-target genes with functions beyond redox regulation—including feather development—while enabling significant metabolic rate increases that avoid trade-offs with lifespan. The strategy of NRF2 activation sought by intense clinical investigation therefore appears to have also unlocked a massively successful evolutionary trajectory.

## Abstract (web summary)

Fast metabolisms tend to shorten lifespans by increasing oxidative damage. This study identifies a gene mutation that keeps a key antioxidant response active, possibly allowing Neoaves bird species to avoid the tradeoff between rapid metabolism and longevity that challenges most mammals, including humans.

## Introduction

Studies investigating the evolution of birds (Aves) have focused heavily on feather development and the skeletomuscular adaptations required for flight^1–3^. From a physiological standpoint, flapping flight deserves special attention as the most metabolically intense form of vertebrate locomotion^4^, requiring high levels of oxygen and imposing a significant demand on oxygen transport systems^5,6^. This is reflected in the extensive cardiovascular and pulmonary adaptations in birds relative to mammals^6,7^ and in their higher metabolic activity and oxygen consumption^5,6,8,9^. In vertebrates, a consequence of high oxygen metabolism and intense aerobic activities (such as flight) is the generation of reactive oxygen species (ROS), which can cause extensive damage to cellular function if not counterbalanced by antioxidant enzymes^10–14^. Oxidative stress is therefore a major constraint on animal performance^13–16^, yet the role of ROS in shaping the evolution of birds has often been overlooked. This is striking, since birds display a stunning diversity of flight modes^5^, small body sizes^17^, and clutch and egg sizes^18,19^, all of which may be expected to further disrupt avian redox balance^7,8,13,14,16,20^. How birds overcame these ROS constraints during their successful radiation is unknown.

Given the high metabolic rates of birds and the concomitant oxidative stress challenge, the long-standing rate of living and oxidative damage theories of aging predict faster aging in avian species^21–25^. Yet, birds are well known to exhibit remarkable longevity, or more specifically maximal lifespan^25^. This apparent paradox has led to investigations and continued speculations into adaptive mechanisms to counteract oxidative stress in avian species. These include a potential increase in endogenous antioxidant capacity^7,25^—a theory supported by in vitro and in vivo analyses identifying high resistance to oxidative stress in birds^26,27^. Nevertheless, the evolution of the avian antioxidant response remains poorly understood. Identification of protein-level antioxidant adaptations in birds may therefore allow a greater understanding of how redox balance was maintained during the evolution of birds.

The transcription factor NRF2 (NF-E2-related factor 2) is the major cellular pathway for regulating the Metazoan antioxidant response against intracellular oxidative stress^28–30^. NRF2 works predominantly through binding to an antioxidant response element (ARE) to activate expression of an extensive set of antioxidant and cytoprotective genes, as well as genes involved in inflammation and metabolism^30–32^. Under unstressed conditions, KEAP1 binds NRF2 and targets it for rapid proteasomal degradation through ubiquitination, thereby maintaining NRF2 at relatively low levels in the cell^30^. Under conditions of oxidative stress, ROS and electrophilic molecules can modify reactive cysteine residues on KEAP1, leading to structural changes that enable nuclear translocation and accumulation of NRF2, thus activating its wide-ranging cytoprotective transcriptional program^30^.

In humans, the KEAP1-NRF2 pathway is of intense clinical interest for its role in cancer and chronic disease^31^ and is of particular interest for its association with human aging^22,23^. We reasoned that a comparative genomics approach may elucidate the role of the KEAP1-NRF2 pathway in avian cellular oxidative stress. In this study, we leverage the wealth of avian genomes generated by recent advances in bird comparative genomics to investigate the molecular evolution of avian KEAP1 and NRF2. Given the breadth of NRF2-regulated genes outside of oxidative stress response^32^, we also investigate NRF2 target genes associated with avian traits (GSTA2, feather β-keratins). We demonstrate that a KEAP1 mutation in the Neoavian ancestor disrupted the highly conserved binding and repression of NRF2 by KEAP1, leading to constitutive NRF2 activity, increased NRF2-driven antioxidant enzyme expression, and decreased oxidative stress in wild Neoaves tissues and cells. Our analyses suggest that the loss of KEAP1 has had far ranging consequences for the phenotypic diversification of Neoaves.

## Results

### Loss of NRF2 binding by Neoaves KEAP1

**Fig. 1:**
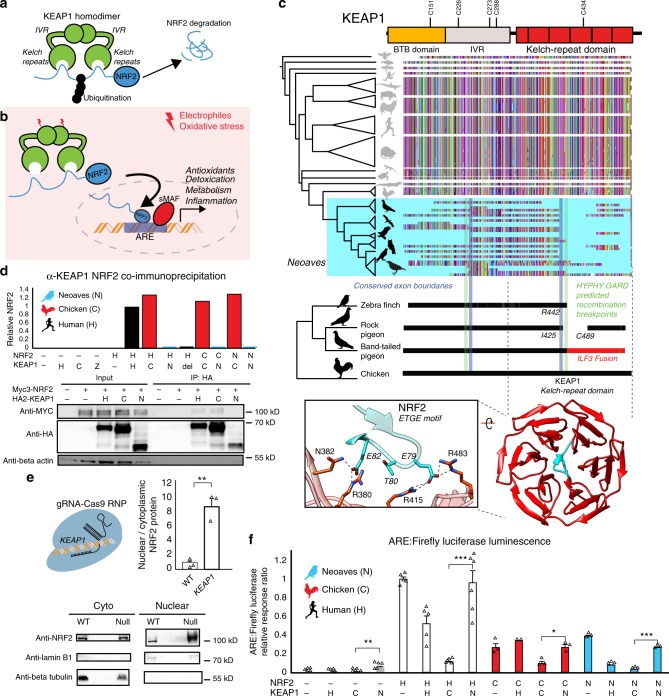
Comparative genomics reveals a loss of KEAP1-NRF2 binding in Neoaves. **a** KEAP1-mediated ubiquitination of NRF2 leads to proteasomal degradation^30^. **b** Under oxidative stress, covalent modification of KEAP1 cysteine sensors trigger NRF2 release and expression of antioxidant response element (ARE)-regulated genes. **c** Alignment of translated  KEAP1 coding sequences obtained from animal genomes. Only fragments of KEAP1 could be retrieved across Neoaves genomes (Supplementary Data File 1). A C-terminal fragmentation pattern includes fusion with neighboring genes (*ILF3*) and coincides with statistically predicted genetic recombination breakpoints (HYPHY, GARD; green lines; “Methods”) that overlap KEAP1 exon boundaries (blue lines). This C-terminal fragmentation pattern affects the sequence integrity of the KEAP1 kelch-repeat domain, which is responsible for binding NRF2 (blue, zoom-in; [PDB ID: 2FLU])^81^. **d** Co-immunoprecipitation of HA-KEAP1 and MYC-NRF2 constructs from transfected HEK293T cell lysates. Relative NRF2/KEAP1 protein levels normalized to that of Human WT demonstrate that both Δ442-488 KEAP1 (del) and Neoaves (N) (*Taeniopygia guttata*) KEAP1 are incapable of binding co-transfected Human (H), Chicken (C), and Neoaves (N; *Zonotrichia albicollis*) NRF2. **e** KEAP1 loss of function (“null”) in Cas9-RNP-treated HEK293T cells leads to increased nuclear NRF2 localization. [*N* = 3; one-sided *t* test, *p* = 0.0013]. **f** Loss of NRF2 binding in Neoaves KEAP1 causes increased transcriptional activity of NRF2, as measured by ARE-driven luciferase expression in Cas9-RNP-treated HEK293T cells transfected with various Human and avian KEAP1 and/or NRF2 constructs [*N* = 6, except for: =2 (C/H); =3 (C/−; C/C; C/N, N/−, N/H, N/C, N/N); =5 (H/−, H/H, H/C); all *N* are biologically independent cells; *p < 0.05; **p < 0.01; ***p < 0.001. Two-sided *t* test]. Source data are provided as a Source data file. All data are presented as mean values. All error bars represent standard error.

We constructed an alignment of KEAP1 coding sequences obtained from publicly available genomes (“Methods”; Supplementary Table 1; Supplementary Data 1). Our final KEAP1 phylogenetic dataset spanned *Drosophila*, teleost fishes, and a detailed sampling of tetrapods (Fig. 1a–c; Supplementary Table 1). The encoded KEAP1 protein sequence is highly conserved between Amphibians, Mammals, and Reptiles and displays strong purifying selection across a wide sampling of mammalian orders (M8 PAML; Supplementary Table 2). KEAP1 is also highly conserved at the base of the Avian phylogeny within the Palaeognathae (*Apteryx rowi*) and the Galloanserae (*Gallus gallus*, *Meleagris gallopavo*, *Anas platyrhynchos*) (Fig. 1c).

In stark contrast to over 300 million years of KEAP1 sequence conservation, we could recover only fragments of KEAP1 coding sequences across 45 Neoaves genomes, altogether representing the work of 15 independent groups employing various sequencing technologies and genome assembly methods (Fig. 1c; Supplementary Data 1; “Methods”). We observed that the fragmentation pattern of Neoaves KEAP1 coding sequences closely corresponded to conserved *KEAP1* exon boundaries in the Chicken (*G. gallus*), Pigeon (*Columba livia*), and Peregrine falcon (*Falco peregrinus*) genome assemblies (Supplementary Data 1; Fig. 1c). Using a genetic algorithm heuristic (GARD HYPHY; “Methods”), we found statistically significant phylogenetic evidence for recombination breakpoints in Neoaves *KEAP1* nearly identical to these conserved exon boundaries (Fig. 1c, green lines; Supplementary Table 3), suggesting an ancestral recombination event in Neoaves *KEAP1*. Consistent with this, within a long-range annotated genome assembly for the band-tailed pigeon (*Patagioenas fasciata*), we found a KEAP1 coding sequence fused at the 3′ end to the adjacent *ILF3* reading frame (accession OPJ82275.1), conjoined precisely at the conserved *KEAP1* exon boundary and predicted recombination breakpoints we detected (R442, Human KEAP1 numbering; Fig. 1c); the noncontiguous remainder of the KEAP1 3′ end was inverted to the antisense strand. This suggested that the fragmentation pattern we observed in Neoaves KEAP1 was due to intrachromosomal rearrangements. To investigate this further, we extracted liver genomic DNA (gDNA) and complementary DNA (cDNA) from the passerine *Zonotrichia albicollis* (White-throated sparrow) and cloned KEAP1 coding sequences from multiple individuals (*n* = 3; “Methods”). Sanger sequencing identified KEAP1 coding sequences lacking the 220 amino acid segment encompassed by the predicted recombination breakpoints, which resulted in a frame-shift at the 3′ end (Supplementary Table 4).

These results provide evidence that the fragmentation pattern we observed (Fig. 1c) represents the loss of a fully contiguous KEAP1 coding sequence through intrachromosomal rearrangement. We hypothesized that this may have been accompanied by a loss of functional constraint on the KEAP1 coding sequence upstream of the R442 breakpoint. Consistent with this, in the recent golden eagle (*Aquila chrysaetos*) chromosome-level genome assembly we found a premature stop codon and frame-shift mutations immediately upstream of the recombination boundary (V377 Human KEAP1 numbering). Within the passerines, we found long branch lengths in the *KEAP1* gene tree and significantly decreased selective constraint relative to the rest of the Avian clade (CmD PAML; “Methods”; Supplementary Fig. 1; Supplementary Table 5). We also found a striking loss of functional residues in Passerine KEAP1 directly involved in mammalian KEAP1 sensing of ROS (C288; ref. ^30^) and cytosolic localization (I304; ref. ^33^), along with the loss of serine residues neighboring S104, which is involved in dimer formation and NRF2 degradation^34^. These residues are otherwise conserved across all vertebrate KEAP1 homologs^28^. Altogether these results suggest that a loss of functional constraint accompanied the loss of a fully contiguous KEAP1 during Neoaves evolution.

We hypothesized that the breakpoint near R442 would disrupt the NRF2-binding domain of Neoaves KEAP1 (Fig. 1c). Within the KEAP1 homodimer, this Kelch-repeat domain is responsible for binding NRF2 at the ETGE and DLG motifs, resulting in the consequent ubiquitination and proteasomal degradation of NRF2 under normal physiological conditions (Fig. 1a; ref. ^30^). Canonically, NRF2 escapes degradation and becomes transcriptionally active only when electrophiles and/or ROS chemically induce conformational changes in KEAP1 via covalent modification of cysteine residues, which leads to NRF2 stabilization and consequent NRF2-mediated activation of a battery of cellular transcription programs (Fig. 1b; ref. ^30^). The loss of this important functional domain in Neoaves KEAP1 therefore suggests that NRF2 is constitutively active within Neoaves. To test this, we used a functioning Human KEAP1 coding sequence capable of binding NRF2 as a template to construct a mutant construct. This mutant Human KEAP1 contained a deletion (Δ442–488) within the NRF2-binding domain of KEAP1 (“Methods”), serving as a conservative representation of the Neoaves fragmentation pattern that starts at the exon boundary/recombination breakpoint near R442 (Fig. 1c; Supplementary Data 1). We also synthesized a predicted Neoaves KEAP1 coding sequence from the Zebra finch genome (*Taeniopygia guttata*; Supplementary Table Data 1), which served as a representative of the wide variety of Neoaves *KEAP1* sequences that lack nearly 200 C-terminal amino acids relative to Chicken KEAP1 starting at R442 (Fig. 1c). Lastly, we directly cloned Chicken KEAP1 from cDNA we synthesized from lung tissue RNA (“Methods”).

We transfected HEK293T cells with constructs containing: Human WT KEAP1; Human Δ442-488 KEAP1 (del); Neoaves KEAP1 (N), or chicken KEAP1 (C) coding sequences (“Methods”). To compare NRF2-binding between these constructs, we co-transfected Myc-tagged NRF2 (“Methods”). We immunoprecipitated HA-tagged KEAP1 from transfected HEK293T cells under conditions favorable to NRF2-KEAP1 binding (“Methods”). In cells co-transfected with either Human or Chicken KEAP1, we observed co-immunoprecipitation (co-IP) of Myc-tagged Human NRF2 (Fig. 1d), as well as both chicken and Neoaves (*Z. albicollis*) NRF2 constructs we cloned from cDNA synthesized from liver tissue RNA (“Methods”; Fig. 1d; Supplementary Fig. 2). By contrast, we found evidence for a complete loss of NRF2 binding by Human Δ442-488 KEAP1, as well as by Neoaves KEAP1, for any co-transfected Human, Chicken, or Neoaves MYC-tagged NRF2 (Fig. 1d; Supplementary Fig. 2). Altogether these bioinformatic and experimental assays indicate that NRF2 binding has been lost in Neoaves KEAP1.

### Loss of KEAP1 binding increases NRF2 activity

We reasoned that a loss of NRF2 binding by Neoaves KEAP1 would result in increased NRF2 transcriptional activity (Fig. 1b). To test this, we conducted NRF2-driven luciferase assays in HEK293T cells. In order to reduce the background effect of endogenous Human KEAP1, we disrupted KEAP1 function in HEK293T cells through CRISPR-Cas9 genome editing (“Methods”; Supplementary Fig. 3). Consistent with a KEAP1 loss of function, we found significantly increased nuclear to cytosolic NRF2 ratios in Cas9-RNP-treated HEK293T cells (Fig. 1e). Furthermore, NRF2 transcriptional activity, target gene expression, and cellular resistance to oxidative stress were also significantly increased (Supplementary Fig. 4; “Methods”). This included a significant increase in *NQO1* expression—an important NRF2-regulated detoxification enzyme^32^ that is pro-tumorigenic via stabilization of hypoxia-inducible factor (HIF)-1^35^. Incredibly, the CRISPR-Cas9-induced *NQO1* overexpression corresponds directly with our observation of a completely deleted *NQO1* locus within Neoaves (Supplementary Fig. 4).

We proceeded to co-transfect these cells with constructs containing Human, Chicken, or Neoaves KEAP1 (as described in the preceding section) along with a firefly luciferase construct under the control of an ARE regulatory element (“Methods”). This allowed us to quantify NRF2 transcriptional activity in response to co-transfected constructs containing KEAP1 coding sequences from different species. We found low levels of luciferase activity in cells co-transfected with either Human or Chicken KEAP1, but in contrast, luciferase expression was significantly increased in cells co-transfected with Neoaves KEAP1 (Fig. 1f), suggesting that the loss of the NRF2-binding domain in Neoaves KEAP1 (Fig. 1c) leads to an increase in NRF2 transcriptional activity. To investigate this further, we co-transfected constructs containing NRF2 coding sequences cloned from Human, Chicken, and Neoaves. We found that NRF2 from all species were transcriptionally active, driving luciferase expression (Fig. 1f). Consistent with our co-IP results (Fig. 1d), Human KEAP1 repressed the transcriptional activity of NRF2 homologs, an effect that was even stronger with Chicken KEAP1 (Fig. 1f). By contrast, we found significantly increased luciferase expression in all cells transfected with Neoaves KEAP1, regardless of the species origin of the co-transfected NRF2 (Fig. 1f). This provides direct evidence that increased Neoaves NRF2 transcriptional activity results from the loss of binding by Neoaves KEAP1.

### NRF2 is functional in vivo within wild Neoaves

**Fig. 2:**
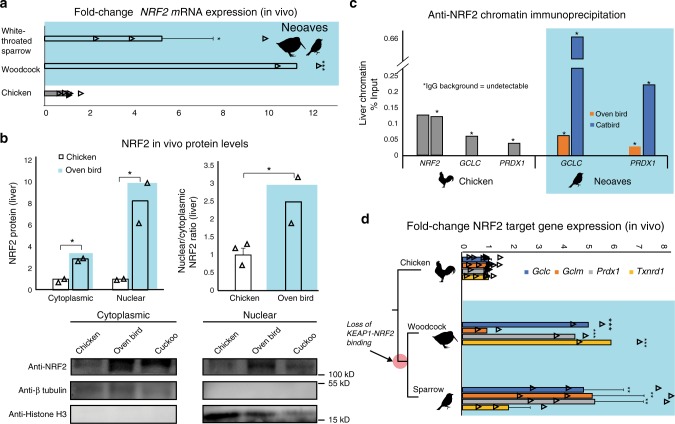
Neoaves NRF2 is functional and upregulated in vivo within wild birds. **a** Relative to Chicken (*Gallus gallus*; [*N* = 6 biologically independent animals]), liver *NRF2* mRNA expression is significantly upregulated in wild Neoaves species (White-throated sparrow *Zonotrichia albicollis* [*N* = 3 biologically independent animals; *p* = 0.014]; American woodcock *Scolopax minor* [*N* = 2 biologically independent animals; *p* = 4.67 × 10^−7^]; one-sided *t* test). All data are presented as mean values. **b** NRF2 protein levels are elevated in both the cytoplasmic and nuclear fractions of wild Neoaves liver lysates (Oven bird *Seiurus aurocapilla* [*N* = 2 biologically independent animals]; Black-billed cuckoo *Coccyzus erythropthalmus* [*N* = 1 biologically independent animal]) relative to Chicken (*Gallus gallus*; [*N* = 2 biologically independent animals]). *p* = 0.0078 and 0.036 for cytoplasmic and nuclear, respectively. One-sided *t* test. Consistent with dysregulation of NRF2 by KEAP1, Neoaves (Oven bird *Seiurus aurocapilla*; [*N* = 2 biologically independent animals]) display increased nuclear to cytoplasmic ratios, relative to Chicken [*N* = 3 biologically independent animals]. *p* = 0.038, one-sided *t* test. **c** Liver chromatin immunoprecipitation using an anti-NRF2 antibody reveals that Chicken and Neoaves (Oven bird *Seiurus aurocapilla*; Gray catbird *Dumetella carolinensis*) NRF2 is bound to antioxidant-response elements (ARE) of *GCLC* and *PRDX1* in vivo. *NRF2* AREs could not be detected in Neoaves genomes, yet two AREs are bound by NRF2 in Chicken liver (note that IgG background is undetectable for all AREs, except for one of the two NRF2 AREs identified in Chicken, where it comprises only 0.6% of signal obtained with the NRF2 antibody; Source data). **d** NRF2 target genes (*GCLC*, *GCLM*, *PRDX1*, *TXNRD1*) are expressed in wild Neoaves (White-throated sparrow *Zonotrichia albicollis* [*N* = 3 biologically independent animals]; American woodcock *Scolopax minor* [*N* = 2 biologically independent animals]) and are significantly upregulated relative to Chicken [*N* = 8 biologically independent animals]. **p < 0.01; ***p < 0.001. One-sided *t* test. Source data are provided as a Source data file. All error bars represent standard error.

With the loss of this critical KEAP1 repressor mechanism in the Neoaves clade, we next investigated NRF2 expression and activity in wild bird tissue (“Methods”; Supplementary Table 6). In order to study *NRF2* mRNA expression, we synthesized cDNA from liver tissue (“Methods”). Through quantitative polymerase chain reaction (qPCR) analysis, we identified *NRF2* transcripts in two highly diverged Neoaves species (White-throated sparrow [*n* = 3] *Z. albicollis*; American woodcock [*n* = 2] *Scolopax minor*) each of which displayed significant NRF2 overexpression relative to that of Chicken (Fig. 2a). We next quantified protein levels of NRF2 in vivo. We extracted cytoplasmic and nuclear fractions from Chicken liver tissue, as well as that from two highly diverged Neoaves species (Oven bird *Seiurus aurocapilla*; Black-billed cuckoo *Coccyzus erythropthalmus*) (“Methods”). Consistent with a non-functional KEAP1 incapable of promoting NRF2 degradation, we found a significant increase in both cytosolic and nuclear NRF2 protein levels in Neoaves relative to that of Chicken (Fig. 2b). In addition, the nuclear to cytosolic ratio of NRF2 was significantly increased in Neoaves relative to Chicken (Fig. 2b). Since this ratio controls for NRF2 expression levels, it provides strong evidence that the increase in nuclear NRF2 in Neoaves relative to Chicken is due to a dysregulation by Neoaves KEAP1. We next investigated whether NRF2 is directly bound in vivo to the AREs upstream of two important NRF2 target genes, *GCLC* and *PRDX1*, which are involved in glutathione- and thioredoxin-based antioxidant systems, respectively^32^. We identified AREs upstream of *GCLC* and *PRDX1* loci within the Chicken and Zebra finch (Neoaves) genomes (Supplementary Table 7; “Methods”) and detected NRF2 occupancy of these regulatory elements in Neoaves (Oven bird *S. aurocapilla*; Gray catbird *Dumetella carolinensis*) and Chicken using anti-NRF2 chromatin immunoprecipitation (ChIP; Fig. 2c; Supplementary Fig. 5). Furthermore, we identified two AREs upstream of the Chicken *NRF2* locus (Supplementary Table 7) and demonstrated NRF2 occupancy of both of these regulatory elements using ChIP (Fig. 2c). Interestingly, we could not detect any AREs upstream of multiple Neoaves *NRF2* loci (Supplementary Table 7), suggesting a loss of NRF2 autoregulation in Neoaves. To test for Neoaves NRF2 transcriptional activity, we investigated the expression of *GCLC* and *PRDX1* mRNAs, along with other important NRF2 target genes (*GCLM*, *TXNRD1*). Consistent with the binding of NRF2 to *GCLC* and *PRDX1* AREs (Fig. 2c), we identified mRNA expression of all four NRF2 targets across two highly diverged wild Neoaves species (White-throated sparrow [*n* = 3] *Z. albicollis*; American woodcock [*n* = 2] *S. minor*), which were significantly overexpressed relative to that of Chicken (Fig. 2d). Altogether these results demonstrate that NRF2 is expressed and functional in vivo within wild Neoaves and strongly suggest a loss of KEAP1 regulation of the NRF2 antioxidant response.

### Constitutive NRF2 nuclear localization in Neoaves cells

**Fig. 3:**
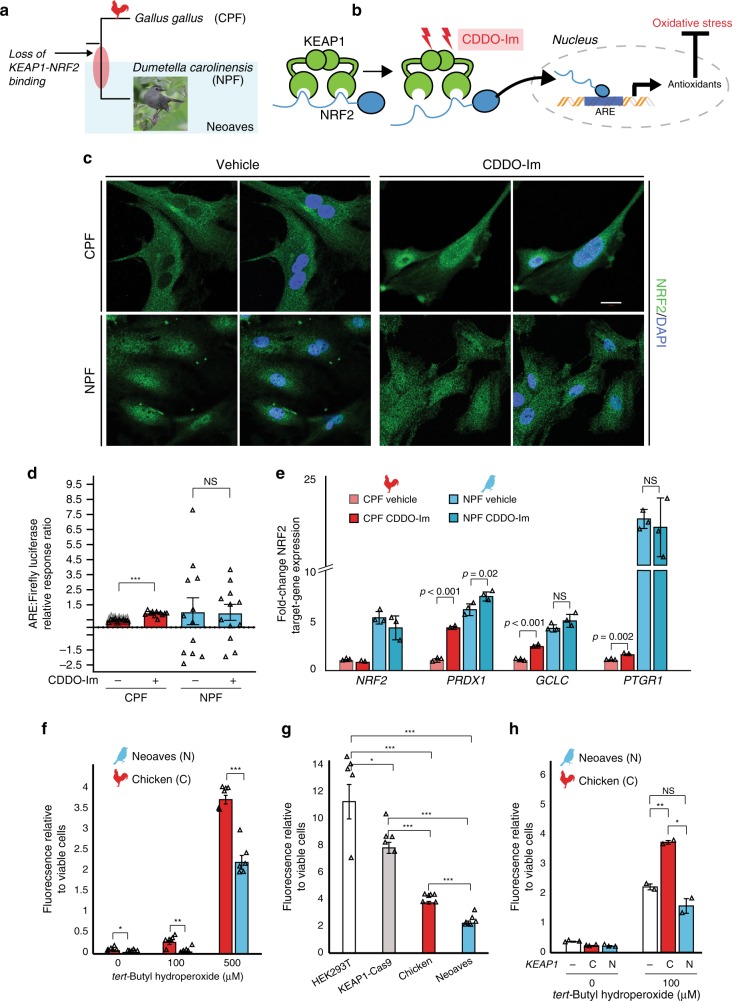
Constitutive NRF2 nuclear localization increases resistance to oxidative stress. **a** Chicken (CPF) and Neoaves (*Dumetella carolinensis*) primary fibroblasts (NPF) were used to evaluate KEAP1 regulation of NRF2 in vitro. “Gray Catbird” [CC BY 2.0] Andy Reago & Chrissy McClarren. https://flickr.com/photos/80270393@N06/17166164877. No changes were made. **b** CDDO-Im is a chemical modulator of KEAP1 that induces release of NRF2 and consequent gene transcription at nuclear antioxidant response elements (AREs; “Methods”). **c** Immunocytochemistry and confocal microscopy (scale bar (white) for all micrographs is 20 µm) of cells exposed to vehicle (DMSO) or 50 nM of CDDO-Im. NRF2 immunofluorescence in CPF and NPF cells. This experiment was repeated independently *N* = 5 times, with similar results. **d** Luciferase assays of vehicle- and CDDO-Im-treated CPF cells [*N* = 10 biologically independent cells; ***p = 5.07 × 10^−12^, one-sided *t* test] and NPF cells [*N* = 12 biologically independent cells]. **e** Multiple NRF2 target genes are significantly upregulated in Chicken cells upon CDDO-Im treatment [*N* = 2 biologically independent cells] relative to vehicle [*N* = 3 biologically independent cells]. These same target genes are constitutively expressed at high levels in Neoaves cells and show no response to CDDO-Im [*N* = 3]. *p* Values are noted above each comparison. One-sided *t* test. **f** Significantly decreased oxidative stress in NPF cells relative to CPF cells when challenged with various concentrations of *tert*-butyl hydroperoxide (tBh) [*N* = 6 biologically independent cells, except for at 500 µM tBh where *N* = 5 biologically independent cells]. 0 μM, *p = 0.045; 100 μM, **p = 0.002; 500 μM, ***p = 1.54 × 10^−5^. Two-sided *t* test. **g** Human cells (HEK293T) with Cas9-induced KEAP1 loss of function display significantly decreased oxidative stress relative to wild type when challenged with 500 μM tBh [*N* = 5 biologically independent cells]. Two-sided *t* test. *(*p* < 0.05); **(*p* < 0.01); ***(*p* < 0.001). This oxidative stress reduction is strikingly similar to that between NPF and CPF cells. **h** Oxidative stress is increased in NPF cells transfected with Chicken KEAP1 (C) relative to those transfected with Neoaves KEAP1 (N; *Taeniopygia guttata*) or mock transfected (−) and exposed to various concentrations of tBh [100 μM, *N* = 2 biologically independent cells; 0 μM, *N* = 3 biologically independent cells]. Mock vs. C, **p = 0.006; Mock vs. N, *p* = 0.14; C vs. N, *p = 0.014; two-sided *t* test. Fluorescence Ex/Em: 492–495/517–527 nm. Source data are provided as a Source data file. All data are presented as mean values. All error bars represent standard error.

Given the in vivo evidence for a loss of KEAP1 regulation of NRF2 in Neoaves, we conducted a series of experiments to investigate this finding under controlled in vitro conditions. We hypothesized that, if Neoaves have indeed lost KEAP1-mediated regulation of NRF2, then relative to Human and Chicken cells, NRF2 in Neoaves cells should display: (1) robust, constitutive nuclear localization and (2) a loss of inducibility by KEAP1 modulators. We used immunocytochemistry to analyze NRF2 immunofluorescence in Chicken primary fibroblasts (CPF) as well as in primary fibroblasts we isolated from a wild Neoaves individual (*D. carolinensis*; NPF; Fig. 3a). We exposed both cell types to a synthetic triterpenoid (CDDO-Im; 2-Cyano-3,12-dioxooleana1,9-dien-28-imidazolide) that induces NRF2 nuclear translocation through structural modification of KEAP1 thiol groups (Fig. 3b; “Methods”). Consistent with a functional and inducible NRF2-KEAP1 system, we found that Chicken NRF2 was strongly localized in the cytosol of CPF cells (Fig. 3c, vehicle) and also displayed a strongly inducible nuclear localization response to CDDO-Im treatment (Fig. 3c). In striking contrast, Neoaves cells displayed strong constitutive NRF2 nuclear localization (Fig. 3c, vehicle), and there was no increase in NRF2 nuclear localization in Neoaves cells treated with CDDO-Im (Fig. 3c, CDDO-Im, bottom row). These results are consistent with the loss of KEAP1-mediated regulation of NRF2 in Neoaves.

To evaluate and quantify the impact of loss of NRF2 regulation by KEAP1 in Neoaves cells, we measured NRF2 transcriptional activity using two approaches: a firefly luciferase construct under the control of an ARE, and by measuring the endogenous expression levels of NRF2 target genes (“Methods”). We found a significant increase in both NRF2-driven luciferase activity and target gene expression in CPF cells treated with CDDO-Im (Fig. 3d, e), providing additional evidence that *G. gallus* has maintained the inducible KEAP1-NRF2 system conserved among other vertebrates^28,30^. This is also consistent with our luciferase experiments in HEK293T cells that demonstrated recombinant Chicken KEAP1 strongly suppresses NRF2 transcriptional activity (Fig. 1f). In contrast, NPF cells treated with CDDO-Im displayed no change in luciferase activity and only small changes in NRF2 target gene expression following CDDO-Im treatment (Fig. 3d, e), consistent with our immunocytochemistry data (Fig. 3c). Furthermore, NRF2 target genes were constitutively expressed at high levels in NPF cells even in the absence of CDDO-Im (Fig. 3e). Since Neoaves NRF2 is indeed capable of driving luciferase expression in HEK293T cells (Fig. 1f) and is expressed and functional in wild-type Neoaves tissues (Fig. 2b–d), our immunocytochemistry, luciferase and gene expression experiments therefore provide strong evidence that an inducible KEAP1-NRF2 system has been lost in Neoaves. This has resulted in constitutive NRF2 nuclear localization and target gene expression.

We hypothesized that the loss of KEAP1 regulation of NRF2 would have strong consequences for cellular resistance to oxidative stress burden. Consistent with this, we found significantly decreased oxidative stress in Neoaves vs. Chicken cells challenged with various concentrations of *tert*-butyl hydroperoxide (tBh; Fig. 3f). To investigate whether this was due to a loss of KEAP1, we employed two complementary experimental strategies. First, we compared the effects of KEAP1 loss of function on oxidative stress using *KEAP1*-Cas9-treated HEK293T cells. As expected, oxidative stress was significantly decreased in KEAP1 loss of function cells relative to wild type, and interestingly, this relative decrease was strikingly similar to that between Chicken vs. Neoaves cells (Fig. 3g). This suggests that a KEAP1 loss of function mediates this difference in resistance to oxidative stress burden. Second, we attempted to rescue KEAP1 repression of NRF2 in Neoaves cells through transfection with Chicken vs. Neoaves KEAP1 constructs. We found that Chicken KEAP1 significantly increased oxidative stress in Neoaves cells, relative to a mock transfected control (Fig. 3h), suggesting repression of endogenous Neoaves NRF2. This is consistent with binding of Chicken KEAP1 to Neoaves NRF2 (Fig. 1d; Supplementary Fig. 2), which represses Neoaves NRF2 transcriptional activity (Fig. 1f). By contrast, transfection of exogenous Neoaves KEAP1 had no effect on oxidative stress in Neoaves cells exposed to 100 μM tBh (Fig. 3h), consistent with the loss of NRF2 binding and repression by Neoaves KEAP1 (Fig. 1d, f). Taken together, these results demonstrate that KEAP1 loss of function in Neoaves has resulted in an increased resistance to oxidative stress.

### Evolutionary trade-offs of constitutive NRF2 activity

**Fig. 4:**
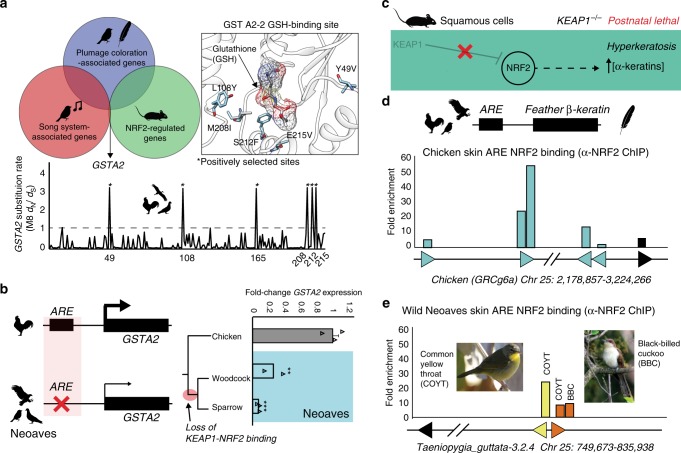
Evolutionary trade-offs of constitutive NRF2 activity in feather development genes. **a** Glutathione *S*-transferase A2 (GSTA2) is shared among gene sets associated with: (1) NRF2-regulation in mice; (2) plumage coloration in Neoaves; and (3) the Zebra finch song system^32, 36, 37^. Avian GSTA2 is a target of positive selection (*p* < 10^−17^; M8 PAML, Supplementary Table 9). GSTA2 sites targeted by natural selection (*d*_N_/*d*_S_ = 3.50) are located within the glutathione (GSH)-binding site (PDB ID: 2WJU) and display Neoaves-specific amino acid variants. **b** Loss of an NRF2 antioxidant response regulatory element (ARE) in Neoaves (Woodcock, *N* = 2 biologically independent animals; Sparrow, *N* = 3 biologically independent animals; Supplementary Table 8) is consistent with a significant decrease in *GSTA2* expression relative to that of Chicken [*N* = 3 biologically independent animals]. **p = 0.005, ***p = 0.0012, respectively, one-sided *t* test. Data are presented as mean values. **c** KEAP1 knockout mice die from starvation shortly after birth from hyperkeratosis of the gastrointestinal tract, likely through overexpression of α-keratins and loricrins in squamous cells^38^. **d** AREs were detected upstream of β-keratins across avian genomes (Supplementary Table 7). Skin chromatin immunoprecipitation (ChIP) with anti-NRF2 antibody revealed NRF2 occupancy of AREs for feather β-keratins (grey) and loricrin (black) identified within the Chicken genome. **e** AREs for feather β-keratins are markedly deceased in the Zebra finch genome (Supplementary Table 7) and display a trend toward decreased NRF2 occupancy in ChIP analysis of the skin obtained from two highly diverged wild Neoaves species (common yellow throat *Geothlypis trichas* and Black-billed cuckoo *Coccyzus erythropthalmus*). “Female Common Yellowthroat” (CC BY-SA 4.0) Sandhillcrane https://commons.wikimedia.org/wiki/File:Female_Common_Yellowthroat.jpg, no changes were made. “Black-Billed Cuckoo” (C) 2008 Wolfgang Wander (GFDL). Source data are provided as a Source data file. All error bars represent standard error.

Our experiments with Cas9 genome editing, along with wild Neoaves tissues and primary cells, provide strong evidence that the loss of KEAP1-NRF2 binding in wild Neoaves reduces the cellular oxidative stress burden through constitutive NRF2 activity. Given the breadth of NRF2-regulated genes^32^, we hypothesized that constitutive NRF2 activity may have also shaped the evolution of important Neoaves-specific physiological traits. We conducted a literature review into potential overlaps between gene sets known to be associated with: (1) NRF2-regulation in mice; (2) plumage coloration in Neoaves; and (3) the Zebra finch song system^32,36,37^. We found that glutathione *S*-transferase A2 (*GSTA2*) is shared among all three gene sets—an enzyme catalyzing the conjugation of reduced glutathione to hydrophobic electrophiles^32^. Given the association of this NRF2 target gene with important Neoaves-specific phenotypic traits, we hypothesized that natural selection may have altered the functional constraints of Neoaves GSTA2, potentially as compensation for the constitutive activation of Neoaves NRF2. We investigated evidence of positive selection on *GSTA2* codons by constructing a large phylogenetic dataset of Avian GSTA2 coding sequences (Supplementary Table 8), followed by *d*_N_/*d*_S_ statistical analyses. We found highly significant evidence of positive selection acting on Avian *GSTA2* (*p* < 10^−17^; M8 PAML, Supplementary Table 9) and identified with high statistical confidence (posterior probabilities >0.99) several GSTA2 sites targeted by this acceleration in evolutionary rates (*d*_N_/*d*_S_ = 3.50; Fig. 4a). Incredibly, all GSTA2 sites under positive selection display Neoaves-specific amino acid variants and are located near the glutathione-binding site within a homology model of the Zebra finch GSTA2 amino acid sequence (Fig. 4a; “Methods”). This is consistent with potential functional adaptation of GSTA2 enzymatic activity, which in turn suggests that the constitutive activation of NRF2 in Neoaves has altered the functional constraints of Neoaves GSTA2.

To further investigate this, we searched for AREs upstream of the *GSTA2* locus in several avian genomes representing a broad sampling of the avian phylogeny (Supplementary Table 8; “Methods”). In contrast to other NRF2 target genes containing an NRF2-bound ARE (GCLC, PRDX1; Fig. 2c; Supplementary Table 7), we could only detect an ARE upstream of Chicken *GSTA2*; we could not find any evidence of an ARE upstream of *GSTA2* across multiple Neoaves species (Fig. 4b; Supplementary Tables 7, 8). Next, we conducted qPCR analysis of *GSTA2* transcripts from liver cDNA synthesized from wild Neoaves liver tissues (“Methods”). We found a highly significant decrease in Neoaves *GSTA2* transcripts in comparison to that of Chicken across a phylogenetically diverse Neoaves species (Fig. 4b), suggesting that the loss of the *GSTA2* ARE in Neoaves has resulted in the downregulation of *GSTA2*. Relative to that of other NRF2 target genes, which contained AREs and were upregulated in Neoaves relative to Chicken (GCLC, PRDX1), the evolution of GSTA2 gene expression therefore appears to have been differentially affected by the constitutive activation of NRF2 in Neoaves. Natural selection therefore appears to have modulated both the GSTA2 protein sequence as well as *GSTA2* expression in Neoaves. Given the unique association between GSTA2 and Neoaves-specific phenotypic traits, we hypothesize that these evolutionary signatures represent compensation for the potentially deleterious effects of constitutive NRF2 activation.

The physiological risks of constitutive NRF2 activation due to loss of KEAP1 binding have been demonstrated in vivo through KEAP1 knockout mice, which die from starvation shortly after birth from hyperkeratosis of the gastrointestinal tract, likely through overexpression of α-keratins and loricrins in squamous cells (ref. ^38^; Fig. 4c). In addition to α-keratins, avian skin keratinocytes also express β-keratin genes, which combine with α-keratins to form avian skin appendages (feathers, scales, claws, beaks; ref. ^3^). We reasoned that if α-keratins are regulated by NRF2 in mouse squamous cells, then it may be the case that β-keratins are regulated by NRF2 in avian skin. We focused on feather β-keratins since they are considered to be expressed at the highest levels compared to other skin appendages^3^. We searched the Chicken genome for ARE regulatory elements upstream of feather β-keratin loci on chromosome 25 (“Methods”; Supplementary Table 7). With loricrin serving as a positive control for our ARE consensus query sequence, we identified multiple feather β-keratins with predicted AREs in this region of the Chicken genome, some of which had multiple AREs per locus (Fig. 4d; Supplementary Table 7). Using chromatin purified from Chicken skin, we detected NRF2 occupancy of these ARE regulatory elements using anti-NRF2 ChIP (Fig. 4d; Supplementary Fig. 5). This suggests that NRF2 may regulate the expression of feather β-keratins within avian skin. We next hypothesized that, similar to the loss of NRF2-mediated ARE-regulation in Neoaves *GSTA2* (Fig. 4b), selection pressures may also be acting to suppress NRF2 regulation of Neoaves β-keratins, potentially as compensation for the constitutively active NRF2 in Neoaves (Fig. 3c). Consistent with this, we detected a marked decrease in ARE elements upstream of β-keratin loci on Zebra finch chromosome 25 (Fig. 4e; Supplementary Table 7), as well as a decrease in NRF2 occupancy at these β-keratin AREs in the skin chromatin of two wild Neoaves diverged across ~80 million of years of evolution (Common yellow throat *Geothlypis trichas* and Black-billed cuckoo *C. erythropthalmus*; Fig. 4e; Supplementary Fig. 5). This strongly suggests that the NRF2-mediated regulation of β-keratins we detected in Chicken skin has been compensated for by the loss of AREs and downregulation of ARE binding by NRF2 at Neoaves β-keratin loci. This pattern closely mirrors the loss of NRF2-mediated ARE-regulation in Neoaves *GSTA2* (Fig. 4b). Together these analyses provide in vivo evidence that the evolution of NRF2-associated feather development genes may have been shaped by the constitutive activation of NRF2 in Neoaves.

### Loss of KEAP1 is associated with increased metabolic rates

**Fig. 5:**
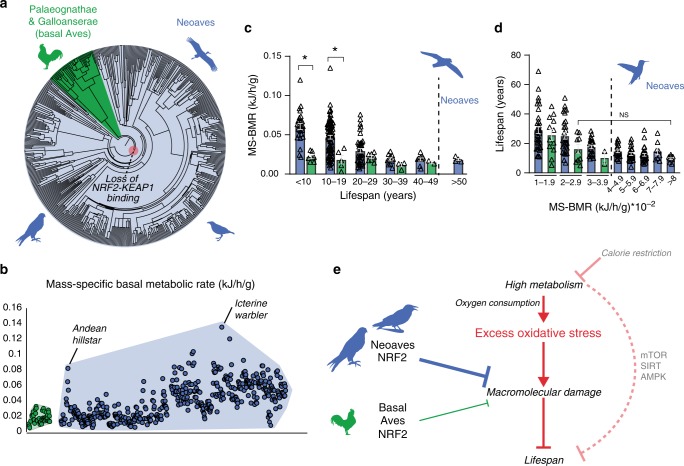
Diversification of lifespan and metabolic rates in Neoaves. **a** Phylogenetic relationships of avian taxa for which mass-specific basal metabolic rates (MS-BMR) are available (“Methods”). Red highlight denotes the Neoavian ancestor where KEAP1 binding of NRF2 was lost. **b** MS-BMR reach higher ranges in Neoaves (blue) species relative to basal Aves (green). Avian orders are grouped by phylogenetic relationships along the *X*-axis (“Methods”). **c** MS-BMR differs significantly between Neoaves and basal Aves species with equivalent lifespans (<10 years, *p* = 0.03; 10–19 years, *p* = 0.02; PI Kruskal–Wallis; Supplementary Fig. 9, Supplementary Tables 10 and 11). Neoaves with low MS-BMR reach higher lifespans than any basal Aves (>50 years). Neoaves: *n* = 180 biologically independent animals; basal Aves: *n* = 25 biologically independent animals. Data are presented as mean values. **d** Neoaves with MS-BMR values higher than that of any basal Aves still maintain statistically indistinguishable lifespans, despite a nearly fourfold increase in MS-BMR (Kruskal–Wallis; Supplementary Table 10). Neoaves: *n* = 175 biologically independent animals; basal Aves: *n* = 23 biologically independent animals. Data are presented as mean values. **e** High metabolism impacts lifespan through nutrient-sensing pathways (mTOR, SIRT, AMPK) as well by generating excess oxidative stress, aggravating age-associated cellular and molecular damage (see “Discussion” for references). An enhanced NRF2 antioxidant response via the loss of KEAP1 would likely lower the risk of macromolecular oxidative damage expected to result from high avian metabolism and oxygen consumption. By altering redox homeostasis to tolerate higher levels of excess oxidative stress, the loss of KEAP1 may have enabled these increases to Neoaves lifespan and metabolic rates. Source data are provided as a Source data file. All error bars represent standard error. * = phylogenetic statistical significance.

We demonstrated that the loss of NRF2 repression by KEAP1 in Neoaves has resulted in increased cellular resistance to oxidative stress burden, which may potentially form an adaptive mechanism capable of counterbalancing high levels of ROS in Neoavian tissues. Since high oxygen consumption and metabolism can produce excess ROS that damage macromolecules, this imposes a key limitation on lifespan^10,12,21–24^. We therefore reasoned that, by altering redox homeostasis, an enhanced NRF2 antioxidant defense may have impacted the evolution of Neoavian metabolism and lifespan. We hypothesized that the loss of KEAP1 would therefore be associated with increases in metabolic rates and lifespan. To investigate this, we obtained publicly available body mass and basal metabolic rate (BMR) data for >530 avian species as well as maximum lifespan data for >1000 avian species (“Methods”). Consistent with our hypothesis, we observed that, across the phylogenetic tree (Fig. 5a), mass-specific BMRs (MS-BMRs) reach higher ranges in Neoaves species relative to that of basal Aves (e.g., ostrich, kiwi, fowl; Fig. 5b).

To quantify these differences, we first controlled for lifespan by stratifying Neoaves and basal Aves species into lifespan groups (Fig. 5c), where within each group the mean lifespans of basal Aves and Neoaves were statistically indistinguishable (Mann–Whitney; Supplementary Fig. 9, Supplementary Table 11). We then conducted a non-parametric statistical analysis on MS-BMR data for species falling into KEAP1 functional (basal Aves) vs. non-functional categories (Neoaves) (Supplementary Table 10). To evaluate statistical significance, we corrected for the effects of phylogeny^39^ by constructing a time-calibrated avian phylogeny for the >200 avian species for which both MS-BMR and lifespan data were available (Supplementary Fig. 9A, “Methods”). We simulated continuous character evolution across the phylogeny to generate empirical null distributions of Kruskal–Wallis (KW) *H* values, which we used to evaluate the statistical significance of KW tests, comprising phylogenetically independent (PI) KW test (“Methods”). Using the presence of a functional KEAP1 as the categorical factor in our model, we found a significant increase in MS-BMR between Neoaves and basal Aves within two different lifespan groupings (<10 years, *p* = 0.03; 10–19.9 years, *p* = 0.02; Supplementary Table 10), providing phylogenetic evidence that the loss of KEAP1 is associated with increased metabolic rates in Neoaves. Furthermore, despite displaying nearly identical MS-BMR values to that of the basal Aves, multiple Neoaves species can reach maximum lifespans beyond the range of any basal Aves in this dataset (Fig. 5c; >50 years). To further investigate this, we stratified all species into MS-BMR-based groups, where the mean MS-BMR of basal Aves and Neoaves were statistically indistinguishable within each group (Mann–Whitney; Supplementary Fig. 9; Supplementary Table 11). Across all MS-BMR groups, there is a clear trend of higher lifespan ranges in Neoaves compared to that of basal Aves (Fig. 5d). Furthermore, even when comparing basal Aves to Neoaves species with nearly fourfold increased metabolic rates (2–2.9 vs. >8 MS-BMR, respectively), there are no significant decreases to Neoaves lifespan (Fig. 5d). This suggests that the constraints imposed on lifespan by MS-BMR (Fig. 5e) have been relaxed in Neoaves, potentially explaining why significant increases to MS-BMR have had no discernable trade-off effect on lifespan during the Neoavian radiation (Fig. 5c, d). Since our analysis presents statistical evidence that the loss of KEAP1 was associated with significant increases to metabolic rates, this raises the intriguing hypothesis that constitutive NRF2 activity may have facilitated the simultaneous diversification of Neoavian MS-BMR and lifespan. As we discuss below, it will be of critical importance to directly investigate this hypothesis using future experimental models.

## Discussion

Bird genomes are characterized by lineage-specific gene loss and intrachromosomal rearrangements that can covary with adaptive responses to environmental challenges and lifestyles^40,41^. This is consistent with our finding that intrachromosomal rearrangements likely caused the loss of a fully contiguous KEAP1 coding sequence in Neoaves. Our in vitro and in vivo analyses reveal that this resulted in a constitutively activated NRF2 that increases resistance to oxidative stress. The loss of a functional KEAP1 in Neoaves appears to have induced compensatory mutations in NRF2 target genes with functions beyond redox regulation—including feather development—while enabling significant increases to metabolic rates with minimal lifespan trade-offs. Here we discuss the relevance of these findings for avian evolution, longevity, and human disease.

The role of oxidative stress in mediating life-history trade-offs has been studied extensively among extant avian taxa^13–16,20,24^. Yet, the importance of ROS in constraining the ancient diversification of birds has received comparably little attention. This is striking, since it has long been appreciated that the high metabolic and oxygen demands of birds (such as for flapping flight^5,6^) implies a significantly greater potential for ROS formation^7,8,14^. Unless countered by antioxidant enzymes, excess ROS induces detrimental oxidative modifications to proteins, DNA, and lipids, disrupting key cellular processes involved in energy metabolism, proteostasis, as well as DNA maintenance (reviewed in refs. ^10,12^). Given the well-documented regulation of multiple antioxidant defense systems by NRF2^30–32^, the robust and constitutive NRF2 activity resulting from loss of KEAP1 repression may have conferred a significant advantage in mechanisms for combatting oxidative damage to cellular macromolecules. These include enzymes that catabolize ROS, as well as reducing factors, notably reduced glutathione, one of the major small molecule antioxidants in cells for counterbalancing ROS production^42^. Our finding of constitutive NRF2 activity in Neoaves is consistent with previous in vitro and in vivo analyses identifying: high resistance to oxidative stress in Neoaves^26,27^, reduced levels of protein and lipid oxidation, DNA damage, and higher expression levels of NRF2 target genes (reviewed in ref. ^8^). Since oxidative stress is a key limitation on animal longevity, physiology, and life-history traits^13–16^, the loss of KEAP1 may have been an important adaptation enabling Neoaves to overcome these constraints during their successful radiation.

Metabolism plays a particularly key role in regulating lifespan through cellular pathways such as nutrient sensing^22,43^, as well as through mitochondrial function and associated redox homeostasis^21–23,43^. The loss of KEAP1 repression of NRF2 and the attendant expected enhancement in antioxidant defense in Neoaves therefore has specific implications for reconciling the long lifespans of birds with their high aerobic metabolism^5–7,14,25^. Since ROS production is directly associated with consumed oxygen^10,12^, it is thought that ROS levels are directly proportional to organismal aerobic metabolic rates (Fig. 5e)^8,24,44^. Excess ROS generated in response to various environmental and cellular conditions can limit lifespan by aggravating age-associated cellular and molecular damage^21–24^. Our data strongly suggest that an enhanced NRF2 antioxidant response via the loss of KEAP1 would likely lower the risk of macromolecular oxidative damage in Neoaves, thus maintaining redox homeostasis^10,12,21,24,30^ otherwise expected to be disrupted by high avian metabolism and oxygen consumption^5–7,14^ (Fig. 5e). We propose that, by altering redox homeostasis to tolerate higher levels of excess oxidative stress, the loss of KEAP1 may have been a key adaptation, permitting increases to Neoaves metabolic rates with minimal lifespan trade-offs, as we detect here. This mechanistic connection between metabolism and lifespan has been recently supported in humans, where metabolic slowing by calorie restriction (Fig. 5e) decreases oxidative damage^45^, and is well known to increase lifespan in model organisms^22,43^. It is also noteworthy that the possible link between the KEAP1-NRF2 system, aging, and lifespan has been investigated in other animal systems. A recent comparative study of ten rodent species demonstrated a close association between the high maximal lifespan potential of naked mole rats and their increased NRF2 signaling, which is due at least in part to significantly lower levels of KEAP1 expression^46^. In *Drosophila*, which possess a KEAP1-NRF2 signaling system for counteracting oxidative stress, experimental loss-of-function mutations in KEAP1 extend lifespan^29^. Similarly, in *Caenorhabditis elegans*, the skinhead-1 SKN-1 homolog of NRF2 can mediate an increase in lifespan likely through activation by mitochondrial ROS^43^.

It is important to discuss potential caveats. Since metabolic rates vary across organ tissues^43^ and environmental conditions^9^, the relationship between BMR and longevity is more complex than what we could address in the present study. Future loss-of-NRF2-function experiments will be necessary to determine the causative role of constitutive NRF2 activity on Neoavian lifespan and metabolism. Second, we observed that fowl (e.g., chicken, ducks, geese) maintain a functional KEAP1, despite the fact that some species (e.g., bar-headed geese) would likely benefit from constitutive NRF2 activity to combat ROS generated by hypoxic high-altitude conditions^47,48^. Similar to Neoaves, there have been rapid radiations across fowl lineages^49^, yet a potentially key difference is the evolution in Neoaves of a stunning diversity of energetically demanding flight modes^5,50^, as well as small body sizes^17^, both of which would be expected to further increase mass-specific aerobic rates and therefore oxidative stress^5–7^. It may be the case that Neoaves were uniquely positioned to evolve these costly phenotypes due to the loss of KEAP1. Evolutionary pathways often include compensatory mutations that permit a complex adaptation, where a non-adaptive mutation deleterious in isolation is required to precede and accommodate the subsequent functional perturbations of the complex adaptation^51^. Indeed, our results suggest that the loss of KEAP1 may have required prior compensatory mutations in Neoaves to overcome the detrimental side effects resulting from constitutive NRF2 activity. Specifically, our results demonstrate that the loss of KEAP1 in human cells leads to a significant increase in *NQO1* expression, which is known to facilitate tumor growth through HIF-1 binding^35^. It is possible that the loss of *NQO1*, as well as *GSTA2* ARE-binding sites, may have been required for the loss of *KEAP1* to be evolutionarily successful. Although speculative, these possible pre-adaptations may have therefore opened an evolutionary trajectory that remains closed to fowl.

The signatures of adaptation we detected in plumage-associated genes (*GSTA2* and β-keratins) indicate that constitutive NRF2 activity may have shaped Neoaves phenotypes other than lifespan and metabolism. For instance, there has been extensive debate surrounding plumage coloration as an honest signal of physiological quality during Neoaves mate choice, with the significant investment of carotenoid antioxidants as one possible basis^52,53^. Associations between reproductive rates, egg size, life-history traits, and oxidative stress have also yielded conflicting findings^8,24^. Our results suggest the loss of KEAP1 may have created a new baseline of enhanced NRF2-driven antioxidant activity in Neoaves, which may help buffer increases in oxidative stress resulting from variation in life-history traits^13–16,20,24^. Similarly, this adaptation may have also permitted carotenoid allocation toward plumage coloration to circumvent deleterious trade-offs with putative antioxidant-related functions, consistent with recent research which shows that carotenoid pigments have little effect on avian antioxidant defenses^54^. Experimental in vivo manipulation of NRF2 in a plumage coloration, feather development, and life-history analysis will be of great interest.

The current investigation also has great relevance for human systems. Appreciation of the impact of oxidative stress and inflammation on chronic disease has spurred strong interest in developing drugs that inhibit KEAP1 binding and repression of NRF2^31^. Numerous studies have demonstrated therapeutic benefit of KEAP1-targeting compounds in multiple experimental models of chronic disease, including respiratory, gastrointestinal, cardiovascular, neurodegenerative, autoimmune, and metabolic diseases. One drug, DMF, has been approved by the Food and Drug Administration and European Medicines Agency for treatment of relapsing multiple sclerosis and psoriasis, and additional compounds are in clinical development that are geared toward chronic suppression of KEAP1^31^. Beyond disease, the link between age-related decline in NRF2 activity and the cellular and molecular hallmarks of aging^22^ have been rigorously examined, leading to speculation regarding the utility of NRF2-based therapies to slow aging^23^. Studying the relationship of KEAP1 to aging in birds and other organisms may provide insights into human aging and approaches to slow this process.

## Methods

### Bird sampling

Neoaves individuals were collected by the Baltimore Bird Club chapter of the Maryland Ornithological Society under a US Fish and Wildlife Salvage Permit (MB-197741-0) during October 2018 and May 2019. Species identifications for birds investigated in this study were provided by the Baltimore Bird Club. Liver, skin, and muscle samples were dissected from Neoaves specimens under Biosafety Level 2 conditions following Johns Hopkins Biosafety protocols. Chicken tissues were collected immediately after slaughter from a local farm (Locust Point Farms, Elkton MD). All October 2018 tissues were immediately stored in RNAlater (Qiagen), whereas May 2019 tissues were snap frozen in liquid N_2_ and stored at −80 °C. gDNA was extracted from avian muscle tissue using the DNeasy Blood and Tissue Kit (Qiagen). Total RNA was isolated using the RNeasy Mini Kit (Qiagen), and single-stranded cDNA was synthesized using MMLV Reverse Transcriptase (Invitrogen).

### Dataset assembly

All KEAP1 coding sequences used in phylogenetic analyses were obtained from Genbank. Mammalian KEAP1 coding sequences (Supplementary Table 1) were obtained through a tBLASTN search against the nucleotide collection (nr/nt) database using the Human KEAP1 coding sequence as query (XM_011528452.1). Non-avian reptile KEAP1 coding sequences (Supplementary Table 1) were obtained through a tBLASTN search of Genbank using *Anolis carolinensis* KEAP1 as a query (XP_003216447.1). The teleost KEAP1 paralogs (KEAP1a/b; ref. ^55^) were obtained directly from the zebrafish genome (*Danio rerio*; Supplementary Table 1). Similarly, KEAP1 coding sequences were obtained directly from the genomes of *Latimeria chalumnae* and *Xenopus tropicalis* (Supplementary Table 1). Avian genomes (Supplementary Data 1) were individually interrogated for KEAP1 coding sequences with tBLASTN searches using Mallard KEAP1 as query (AND99337.1; *A. platyrhynchos*). We determined a conservative cutoff of 70% sequence identity to Mallard KEAP1 when assigning tBLASTN matches from Avian genomes as bona fide KEAP1 coding sequences (Supplementary Data 1). This was based off tBLASTN searches using Mallard KEAP1 as query against the Human (GRCh38.p12 Primary Assembly) and Xenopus genomes (Xenopus_laevis_v2 reference Annotation Release 100), which revealed an 85% and 75% Mallard KEAP1 coding sequence identity to the Human and xenopus *KEAP1* loci, respectively. To more comprehensively search for putative Neoaves KEAP1 coding sequences, we conducted a BLASTN search with the predicted KEAP1 coding sequence from the rock pigeon genome (XM_005515118.1; *C. livia*). This increased coverage of KEAP1 coding sequences from several Neoaves genomes. All KEAP1 coding sequences described above were aligned using PRANK codon alignment, followed by manual adjustment^56^. The final *KEAP1* coding sequence alignment encoded for KEAP1 amino acid residues 56–613 (Human numbering). We used this alignment to construct a *KEAP1* gene tree (IQ-tree; ref. ^57^) using the paralog of KEAP1 (Teleost KEAP1a) as an outgroup. A substitution model was auto-selected, with an ultrafast bootstrap analysis (1000 bootstrap alignments) and an SH-aLRT branch test with 1000 replicates. The identity of all KEAP1 sequences was confirmed by this phylogenetic analysis, which identified all KEAP1 coding sequences as monophyletic, and recapitulated major phylogenetic clades such as mammals and Neoaves (Supplementary Fig. 1). Additional KEAP1 coding sequences were obtained through PCR amplification with a high-fidelity DNA polymerase (Platinum SuperFi, Invitrogen) using *Z. albicollis* gDNA and cDNA, followed by direct Sanger sequencing (Johns Hopkins Genetic Resources Core Facility).

All GSTA2 coding sequences used in phylogenetic analyses were obtained from Genbank (Supplementary Table 8). We constructed an avian GSTA2 coding sequence dataset by performing a tBLASTn search of avian genomes using the predicted Zebra finch GSTA2 (NP_001232758.1) protein sequence as query. To identify bona fide avian GSTA2 from these results, we added Mammalian, Chicken, and *A. carolinensis* GSTA2, GSTA3, and GSTA4 coding sequences along with GSTP1 outgroups and aligned all sequences using PRANK codon alignment, followed by manual adjustment in MEGA10^56^. Using this alignment, we constructed a gene tree using IQ-TREE^57^, where substitution model was automatically selected. Branch support analysis consisted of 1000 bootstraps, along with 1000 SH-aLRT branch test replicates. Avian GSTA2 formed a monophyletic clade sister to mammalian GSTA2 (Supplementary Fig. 6).

### Structural modeling

The protein crystal structures of KEAP1 bound to NRF2 peptide (PDB ID: 2FLU) and GSTA2 (PDB ID: 2WJU) were visualized with the UCSF Chimera package (v1.13.1)^58^. Chimera is developed by the Resource for Biocomputing, Visualization, and Informatics at the University of California, San Francisco (supported by NIGMS P41-GM103311). The structure of Neoaves (Zebra finch *T. guttata*) GSTA2 was inferred via homology modeling using the MODELLER interface in the UCSF Chimera package^59^. The run with the lowest discrete optimized protein energy score was assessed^60^. ProCheck was used to verify the high probability of bond angle and length stereochemical conformations, as indicated by positive overall *G*-factor^61^. Comparisons of each model’s total energy to that expected by random chance were examined using ProSA-web^62^.

### Construct design

Human KEAP1 construct (pcDNA3-HA2-Keap1) was a gift from Yue Xiong (Addgene plasmid # 21556; http://n2t.net/addgene:21556; RRID:Addgene_21556)^63^. The Human KEAP1 deletion mutant (Δ442-488) was created and cloned into the pcDNA3-HA2 backbone through the NEBuilder® HiFi DNA Assembly protocol using a high-fidelity DNA *Pfu* polymerase (Agilent). The complete coding sequence of Chicken (*G. gallus*) KEAP1 was amplified from liver cDNA with a high-fidelity DNA polymerase (Platinum SuperFi, Invitrogen) (Supplementary Tables 6 and 12) and cloned into the pcDNA3-HA2 backbone (NEBuilder® HiFi DNA Assembly Cloning Kit). The predicted Zebra finch (*T. guttata*) KEAP1 coding sequence (NW_002221036.1) was synthesized as a gBlock Gene Fragment (Integrated DNA Technologies) and cloned into the pcDNA3-HA2 backbone (NEBuilder® HiFi DNA Assembly Cloning Kit). Human NRF2 construct (pCDNA3-Myc3-Nrf2) was a gift from Yue Xiong (Addgene plasmid # 21555; http://n2t.net/addgene:21555; RRID:Addgene_21555)^63^. The complete coding sequences of Chicken (*G. gallus*) and White-throated sparrow (*Z. albicollis*) NRF2 were amplified from lung and liver cDNA, respectively (Supplementary Tables 6 and 12) using a high-fidelity DNA *Pfu* polymerase (Agilent) and were then cloned into the pcDNA3-Myc3 backbone (NEBuilder® HiFi DNA Assembly Cloning Kit).

### HEK293T cell culture and NRF2-KEAP1 immunoprecipitation

HEK293T cells were cultured at 37 °C 5% CO_2_ in 100-mm dishes containing minimum essential medium (Gibco) supplemented with 1× GlutaMAX (Gibco) and 10% heat-inactivated fetal bovine serum (Corning). At 85% confluency, cells were transfected with HA2-KEAP1 and/or Myc3-NRF2 plasmid constructs using the Lipofectamine 2000 Transfection Reagent (Thermo Fisher Scientific). Twenty-four hours post-transfection, cells were treated with proteasomal inhibitor MG-132 (Sigma Aldrich). Cells were then lysed 4 h later with a IGEPAL CA-630 lysis buffer (50 mM Tris-HCl [pH 7.5], 150 mM NaCl, 50 mM NaF, 1 mM Na_3_VO_4_, 0.1% IGEPAL CA-630, 1 mM dithiothreitol [DTT], 1× protease inhibitor cocktail). Cell lysates were incubated with monoclonal anti-HA conjugated magnetic bead slurry (MBL) overnight at 4 °C on an inverting rotator. Lysates were washed three times in wash buffer (TBS, 0.1% IGEPAL CA-630, 1 mM DTT), and immunoprecipitated HA2-tagged KEAP1 was eluted by adding 1× Laemmeli buffer, boiling for 5 min, followed by physical separation from magnetic bead slurry using a magnetic rack (MBL). HA2-KEAP1 and/or Myc3-NRF2 proteins were visualized and quantified through sodium dodecyl sulfate-polyacrylamide gel electrophoresis (SDS-PAGE) and immunoblotting.

### qPCR and western blot analysis

Total RNA was isolated using the RNeasy Mini Kit (Qiagen) and single-stranded cDNA was synthesized using MMLV Reverse Transcriptase (Invitrogen). qPCR was performed using the SYBR Green PCR Kit (Qiagen) with a StepOnePlus real-time PCR system (Applied Biosystems). qPCR primers are described in Supplementary Table 12. For quality control, cDNA from wild Neoaves bird specimens used in qPCR analyses were required to display equivalent GAPDH expression levels to that of Chicken. All measurements were taken from biologically independent animals. Likewise, for western blot analysis, Neoaves species chosen were those displaying equivalent housekeeping protein expression in cytoplasmic and nuclear lysate fractions, as determined by a colorimetric assay (Bio-Rad). For co-IP studies, anti-HA (C29F4 Cell Signaling Technology; 1:1000), anti-Myc (71D10 Cell Signaling Technology; 1:1000), and anti-beta actin (13E5 Cell Signaling Technology; 1:1000) antibodies were used. For analysis of NRF2 nuclear translocation, nuclear extracts from HEK293T cells and bird liver samples were prepared using NE-PER Nuclear and Cytoplasmic Extraction Reagents (Thermo Fisher Scientific). For HEK293T cell lysates, anti-NRF2 (16396-1-AP, Proteintech Group; 1:5000), anti-KEAP1 (D6B12 Cell Signaling Technology; 1:1000), and anti-Lamin B1 (D4Q4Z Cell Signaling Technology; 1:1000) antibodies were used. For bird liver cell lysates, anti-NRF2 (16396-1-AP, Proteintech Group; 1:5000), anti-β-tubulin (9F3 Cell Signaling Technology; 1:1000), and anti-Histone H3 (D1H2 Cell Signaling Technology; 1:2000) antibodies were used. The band intensity was quantified using the Image J program (version 1.52d). All measurements were taken from distinct samples.

### NRF2 occupancy of avian AREs

We searched avian genomes for AREs within ~1000 bp upstream of NRF2 target genes using the de novo human consensus motif (TGASWMAKCN) identified by Chorley et al.^64^ (Supplementary Table 7). Primers were designed to amplify 25–50 bp on each side flanking the ARE (Supplementary Table 12). Chromatin was purified from liver samples from wild Neoaves (Oven bird *S. aurocapilla*; Gray catbird *D. carolinensis*) and Chicken. ChIP assay protocol was modified from the Magna ChIP G Tissue Kit manual (17-20000, Millipore). Thirty milligrams of liver or skin tissues were cut into small pieces on ice and washed with phosphate-buffered saline (PBS) with 1× proteinase inhibitor cocktail once and then centrifuged at 5000 rpm. The tissue pellets were cross-linked with 1% formaldehyde at room temperature for 10 min, and the reaction was stopped by adding 0.125 M glycine. The tissue pellets were disaggregated by microgrinder and pestle and were lysed in tissue lysis buffer (5 mM PIPES, pH 8.0, 85 mM KCl, 0.5% NP40, 1× proteinase inhibitor cocktail) by incubating on ice for 15 min. After spin down, the pellets were re-suspended in nuclei lysis buffer (50 mM Tris-HCl pH 8.0, 10 mM EDTA, 1% SDS plus 1× proteinase inhibitor cocktail). After incubation on ice for 20 min, the lysates were subjected to sonication to obtain chromatin DNA fragments ranging from 200 to 1000 bps. The sonicated chromatin was immunoprecipitated by Nrf2 antibody (16396-1-AP, Proteintech Group; 1:250) and the isotype IgG control (30000-0-AP, Proteintech Group; 1:250) and captured on protein G magnetic beads (Millipore). Bound chromatin was then washed in low-salt wash buffer (20 mM Tris-HCl, pH 8.0 150 mM NaCl, 2 mM EDTA, 1% Triton X-100, and 0.1% SDS), high-salt wash buffer (20 mM Tris-HCl, pH 8.0, 500 mM NaCl, 2 mM EDTA, 1% Triton X-100, and 0.1% SDS), LiCl wash buffer (10 mM Tris-HCl, pH 8.0, 250 mM LiCl, 1 mM EDTA, 1% NP-40, and 1% SDS), and TE buffer and subjected to elution, de-crosslinking, RNase and Proteinase K treatment in elution buffer (1% SDS and 50 mM NaHCO_3_) followed by spin column purification (Qiagen PCR purification kit).

### CRISPR-Cas9 genome editing

Predesigned Alt-R CRISPR-Cas9 crRNA targeting the Human *KEAP1* locus, along with Alt-R CRISPR-Cas9 tracrRNA, were obtained from Integrated DNA Technologies (Supplementary Table 13). Predesigned negative and positive control crRNAs were obtained from the Alt-R CRISPR-Cas9 Human Control Kit (Integrated DNA Technologies). Guide RNA (gRNA) was prepared from crRNA and tracrRNA at 100 μM final concentration in Nuclease-Free Duplex Buffer (Integrated DNA Technologies) according to the manufacturer’s protocol. Separate ribonucleoprotein (RNP) complexes consisting of Alt-R S.p. HiFi Cas9 Nuclease V3 (Integrated DNA Technologies) and a single gRNA were formed in Opti-MEM media (Thermo Fisher Scientific) with Cas9 PLUS reagent (Thermo Fisher Scientific). RNP complexes at a final concentration of 50 nM were reverse-transfected into HEK293T cells (ATCC® CRL-3216™) using CRISPRMAX transfection reagent (Thermo Fisher Scientific). Cells were incubated at 37 °C, 5% CO_2_ for 48 h.

We used the Alt-R Genome Editing Detection Kit (Integrated DNA Technologies) to determine off-target genome editing of the KEAP1 gRNA, as well as to estimate the editing efficiency of RNPs containing KEAP1 gRNA and control gRNAs. Briefly, gDNA was isolated from RNP-treated and untreated HEK293T cells using the DNeasy Blood and Tissue Kit (Qiagen). Off- and on-target loci containing PAM sequences computationally predicted (Integrated DNA Technologies) to be targeted by our KEAP1 crRNA (Supplementary Table 13) were amplified with a Taq polymerase (Promega), using primers manually designed to amplify 1000 bp fragments with >100 bp flanking the PAM sequence (Supplementary Table 12). Amplicons were then digested by T7 endonuclease I (T7EI), and results were analyzed by gel electrophoresis (Supplementary Fig. 3). Sensitivity of the T7EI assay was validated using control oligonucleotides (Integrated DNA Technologies). Editing of the *KEAP1* locus was separately confirmed by generating amplicons using a *Pfu* high-fidelity polymerase (Agilent) followed by direct Sanger sequencing (Johns Hopkins Genetic Resources Core Facility). We extracted total cell lysate using the IGEPAL CA-630 lysis buffer described above, as well as the cytoplasmic and nuclear fractions (NE-PER Kit, Thermo Fisher Scientific) of RNP-treated vs. untreated HEK293T cells, and quantified KEAP1 and NRF2 protein levels in these lysates through SDS-PAGE and immunoblotting. All measurements were taken from distinct samples.

### Luciferase assays

Cas9-RNP-treated HEK293T were seeded into 96-well plates at a density of 10,000 cells. Twenty-four hours later, cells were then co-transfected (Mirus TransIT-X2 Dynamic Delivery System) with various NRF2 and KEAP1 constructs, Renilla (pRLS), and Firefly (pGL3 or hQR41) luciferase constructs, in which hQR41 firefly luciferase is under the control of an ARE from Human NQO1^65^. Twenty-four hours post-transfection, cells were treated with CDDO-Im, a potent KEAP1-mediated activator of NRF2^66–68^. Twelve hours later, luciferase luminescence was quantified using a commercial kit (Promega Dual-Glo® Luciferase Assay System) and a Luminometer (BMG Labtech). Relative response ratios were calculated using a scale factor derived from the difference between the Renilla-normalized firefly luminescence of Human NRF2 co-transfected with hQR41 vs. pGL3 transfected cells. All measurements were taken from distinct samples.

### Avian primary fibroblasts

Primary cell cultures were established from a wild Neoaves species (*D. carolinensis*). A 5 × 5 mm^2^ piece of skin was excised, washed with a beta-iodine antiseptic solution, rinsed with PBS three times, and placed into cold culture media (Dulbecco’s modified Eagle medium [DMEM], high-glucose variant [4.5 mg/mL] with sodium pyruvate, supplemented with 10% heat-inactivated fetal bovine serum [Corning], 2% Chicken serum [Gibco], 10 mM HEPES, and 1× antibiotic-antimycotic [Gibco]). Skin was then exposed to 0.5% Collagenase type I solution overnight at 37 °C 5% CO_2._ Digested skin tissues were then seeded into T-25 culture flasks and grown at 37 °C 5% CO_2_ until 90% confluency. Primary fibroblasts were then expanded through passaging in T-75 culture flasks, harvested, and cryopreserved in DMEM supplemented with 40% heat-inactivated fetal bovine serum and 10% dimethyl sulfoxide (DMSO). Cells were stored in liquid N_2_ for up to 4 months prior to assays. CPFs were obtained commercially (Charles River) and cultured and expanded as described above.

### Oxidative stress assays

ROS production was quantified by the dichlorofluorescein (DCF) assay (Invitrogen). Cas9-RNP-treated and untreated HEK293T cells were seeded into 96-well plates at 15,000 cell density per well. Primary dermal fibroblasts isolated from a wild Neoaves species (*D. carolinensis*) were seeded into 96-well plates at 5000 cell density per well. At 80% confluency, media was either removed or cells were transfected with Chicken and Neoaves KEAP1 constructs (Mirus TransIT-X2 Dynamic Delivery System), followed by media removal 48 h later. Cells were treated with 10 µM CM-H2DCFDA (Invitrogen) for 30 min, followed by treatment with or without different doses of tBh (Sigma) for 60 min. DCF fluorescence was measured with a spectrophotometer (BMG Labtech). Cell viability was determined subsequently through a luminescent ATP quantification (Promega) read by a luminometer (BMG Labtech). All measurements were taken from distinct samples.

### Immunocytochemistry

Cells were fixed with 4% paraformaldehyde for 20 min and washed with PBS. After being permeabilized with 0.5% Triton X-100 in PBS for 5 min, the cells were washed for 3 times with PBS. Cells were then blocked with 4% bovine serum albumin with PBS plus 0.05% Tween 20 (PBST), and incubated with anti-NRF2 antibody (16396-1-AP, Proteintech Group; 1:50) for 1 h at room temperature. Cells were washed with PBST and then were incubated with anti-rabbit IgG conjugated with Alexa fluor 488 (Invitrogen, 1:1000), anti-rabbit IgG conjugated with Alexa fluor 594 (Invitrogen, 1:1000), or anti-rat IgG conjugated with Alexa fluor 488 (Invitrogen, 1:1000). DAPI (Vector Laboratories) was used to stain nuclei. Images were taken with a Zeiss LSM 710 confocal microscope. Cells were exposed to CDDO-Im or DMSO vehicle for 16 h before fixation.

### Statistical analysis

We used codon-based likelihood models of molecular evolution from the PAML 4.7 software package to characterize the evolutionary rates of mammalian and avian *KEAP1*, as well as avian *GSTA2*. For these analyses, we constructed a species tree using established relationships^69,70^ (Supplementary Figs. 7 and 8). First, we estimated the evolutionary rates (*d*_N_/*d*_S_) within mammalian (*KEAP1*) and avian (*GSTA2*) datasets independently using the random site models (M1, M2, M3, M7, M8) implemented in the CODEML program^71^ (Supplementary Tables 2 and 9). Avian *GSTA2* sites predicted to be in the positive selection site class of M8 were identified by high posterior probabilities produced by Bayes empirical Bayes analysis^72^. To identify evidence of genetic recombination in our avian *KEAP1* dataset, we employed a maximum likelihood method (GARD) implemented in the HyPhy datamonkey server^73,74^. Based on the evidence for recombination (Supplementary Table 3), we created a tetrapod *KEAP1* dataset consisting only of phylogenetically congruous *KEAP1* coding sequence (631–1411, Human *KEAP1* numbering). We analyzed this pruned dataset using PAML Clade model D (CmD) to explicitly test for long-term shifts in evolutionary rates (*d*_N_/*d*_S_) between foreground and background clades within the tetrapod *KEAP1* datasets (Supplementary Table 5; ref. ^75^). In any partitioning scheme, all non-foreground data are present in the background partition. The foreground partitions are listed after the underscore for the clade models (e.g., CmC_*Birds vs. Mammals*). M3 with three site classes was used as the null model for CmD. All random sites and clade model PAML model pairs were statistically evaluated for significance by likelihood ratio tests with a χ^2^ distribution.

We obtained body mass and BMR (kJ/h) data for >530 avian species from a published dataset stringently curated to eliminate sources of variation on BMR (such as inactivity, seasonality, and thermoneutrality^9^). We calculated MS-BMR as the ratio of BMR to mass. We collected maximum lifespan data for >1000 avian species from the Human Ageing Genomic Resources AnAge database^76^. The final dataset prepared for statistical analysis was obtained by cross-referencing both BMR and lifespan datasets, resulting in a final dataset of 206 species. Over 97% of species in this consolidated dataset had lifespan data designated as “acceptable” or “high” by AnAge curators^76^. To increase sample size, we included “questionable” lifespan data points since these reflected conservative estimates of Neoaves lifespan (i.e., values were less than their closest relatives in the same genus or order). Basal Aves and Neoaves species were stratified according to lifespan and MS-BMR intervals. The mean lifespan and MS-BMR in each respective grouping were statistically indistinguishable [Mann–Whitney, Supplementary Table 11; binned MS-BMR and lifespan data was found to be non-normal, even after various transformation attempts (as assessed by a Ryan–Joiner test in Minitab 19)]. We first evaluated statistical significance by conventional KW tests with KEAP1 functional status as the categorical factor in our model (0,1) and either MS-BMR or lifespan data as the response (Minitab 19). Neoaves vs. basal Aves groupings with conventionally significant *p* values (<0.05) were selected for PI statistical analysis. This was conducted via the computer simulation method^77^ using a time-calibrated avian phylogeny we constructed by following relationships and node dates from recent fossil-calibrated phylogenomic studies^70,78^. We constrained branch lengths to these node dates and resolved polytomies through reference to the time tree of life^79^. Using this phylogeny, we conducted 1000 simulations of continuous character evolution using a model of Brownian motion in MESQUITE^80^. We used these simulations to generate empirical null distributions of KW *H* values through manual analysis in Minitab 19, ensuring the simulated characters originated from the same phylogenetic position as the species analyzed within a given KW test. The 95th percentile of the empirical null *H* value distribution was used as the significance threshold for our KW analyses.

### Reporting summary

Further information on research design is available in the Nature Research Reporting Summary linked to this article.

## Supplementary information

Supplementary Information

Peer Review File

Reporting Summary

Description of Additional Supplementary Files

Supplementary Data 1

## Data Availability

NCBI accession numbers generated by this study are listed in Supplementary Table 4 (MN416129, MN416130, MN416131, MN416132, MN416133). Other NCBI accession numbers generated by previous studies are listed in the Supplementary Data 1, Supplementary Tables 1, 7 and 8. All other data are available in the main text and its supplementary information files. The source data underlying Figs. 1D–F, 2A, B, D, 3D–H, 4B, and 5A, C, D, and Supplementary Figs. 2A, B, 3A–C, 4A, C, D, 5A–I, and 9B, C are provided as a Source data file.

## Source Data

Source Data

## Code availability

All software and associated codes used here were obtained from commercial or published public sources. Detailed descriptions are provided in the “Methods” section.
