# Supplementary material for: Adaptation of the master antioxidant response connects metabolism, lifespan and feather development pathways in birds
Source: Nat Commun. 2020 May 18;11:2476. doi: 10.1038/s41467-020-16129-4 (PMC7234996; doi:10.1038/s41467-020-16129-4)
Supplement: Supplementary file 1 — Supplementary Information [file 41467_2020_16129_MOESM1_ESM.pdf]

Supplementary Information

Adaptation of the master antioxidant response connects metabolism, lifespan and feather development pathways in birds

Castiglione et al.

Supplementary Figure 1

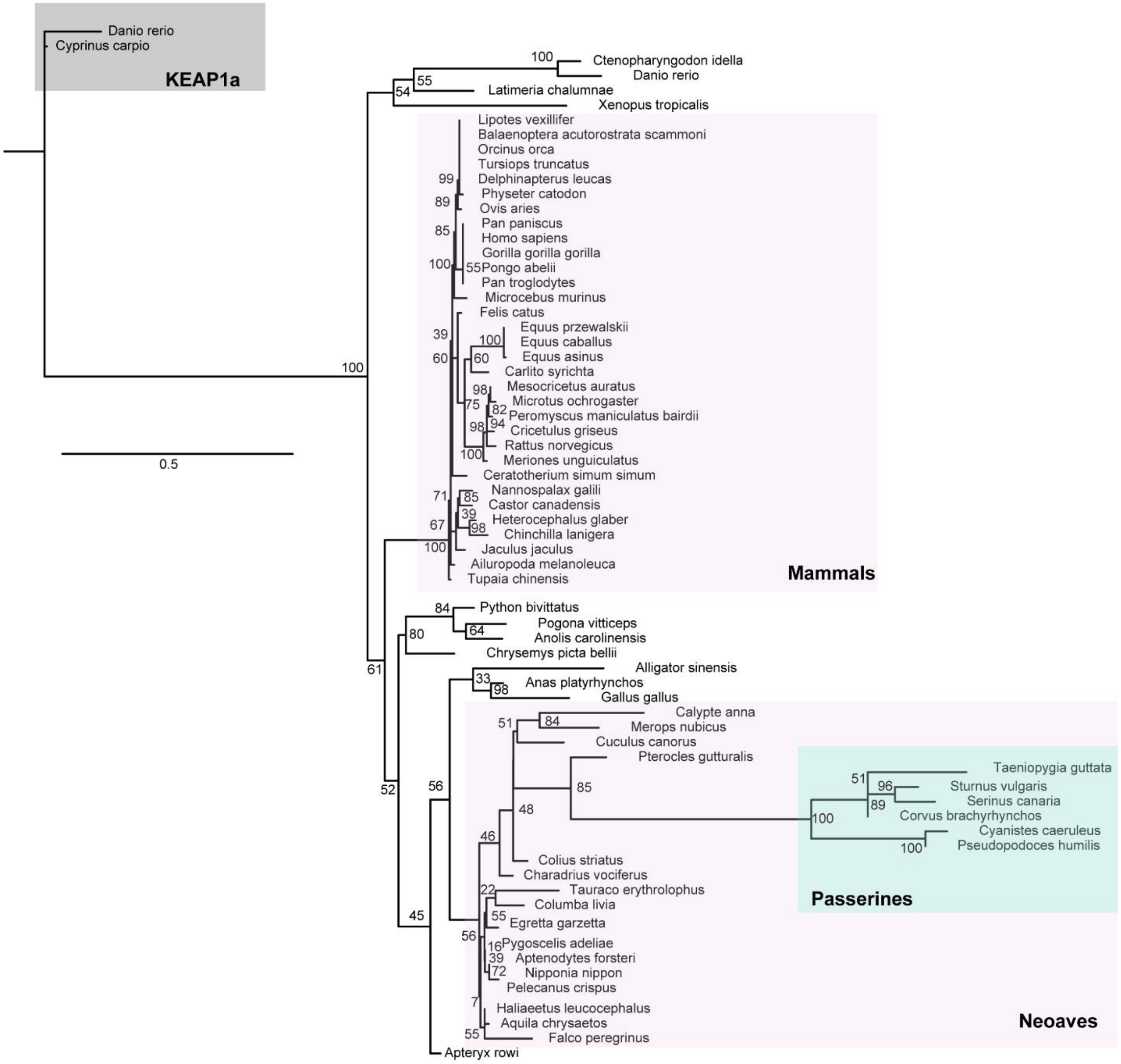

KEAP1 gene tree generated to confirm identity of KEAP1 coding-sequences.

Supplementary Figure 2.

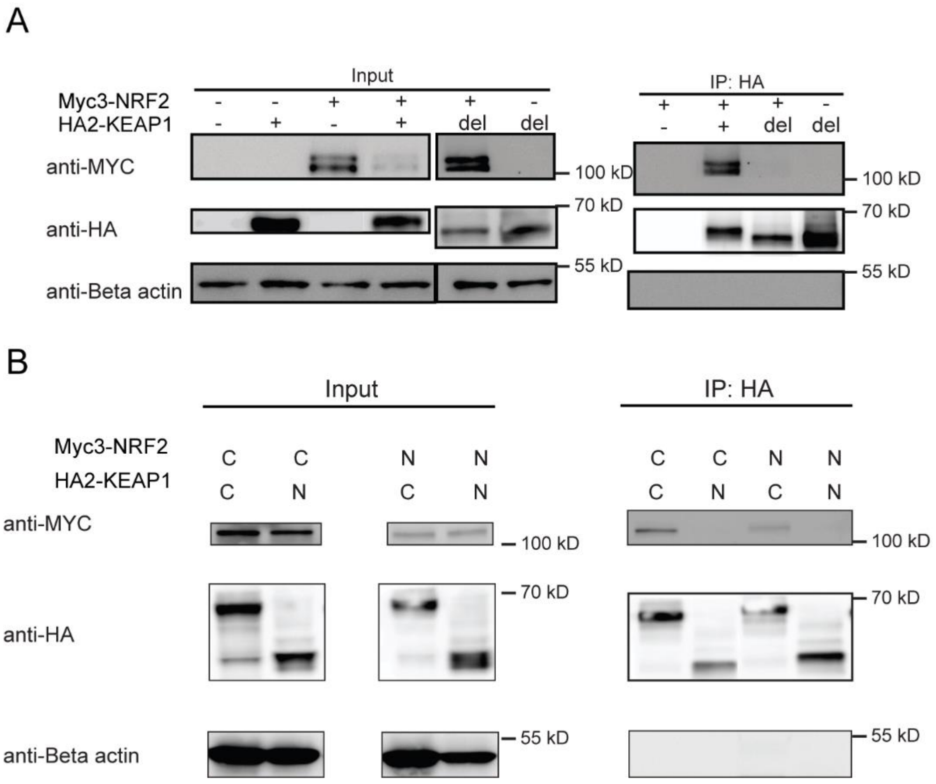

Co-immunoprecipitation of HA2-KEAP1 and Myc3-NRF2 from HEK293T cells transfected with various NRF2 and KEAP1 constructs. (**A**) Human wild-type NRF2 (+) was co-transfected with either wild-type KEAP1 (+) or Δ442-488 KEAP1 (del). (**B**) Chicken (C) or Neoaves (N; *Zonotrichia albicollis*) NRF2 was co-transfected with Chicken (C) or Neoaves (N) (*Taeniopygia guttata*) KEAP1. Source data are provided as a Source Data file. All of these immunoblot experiments were repeated twice each, with each independent replicate showing similar results.

Supplementary Figure 3.

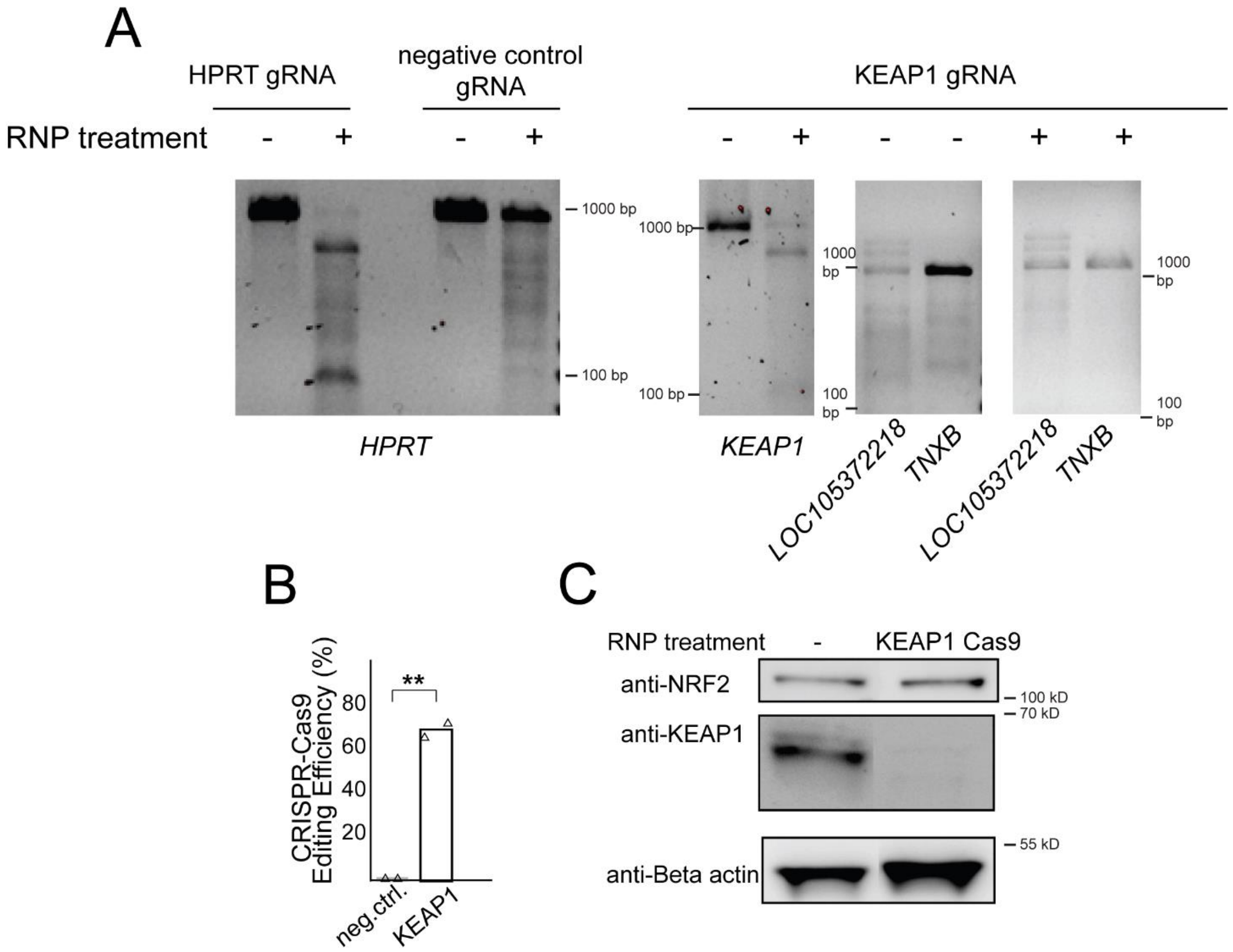

CRISPR-Cas9 genome editing detection. (**A**) Amplicons of control (HPRT), KEAP1, and off-target loci were digested by T7 endonuclease I (T7EI) to evaluate mismatch between annealed DNA strands from genomic DNA isolated from RNP treated HEK293T cells. HPRT gRNA RNP served as positive and negative control for Cas9 editing. KEAP1 gRNA RNP editing efficiency was assessed at both KEAP1 and off-target loci (LOC105372218; TNXB) and results were analyzed by gel electrophoresis. (**B**) Quantification of band intensity from T7EI-digested KEAP1 gRNA RNP-treated HEK293T cell amplicons was performed using the Image J program (version 1.52d) [N=2; p=0.001, one-sided t-test]. (**C**) Western blot of cell lysates from HEK293T cells treated with KEAP1 gRNA Cas9 RNP demonstrates loss of KEAP1 expression. Source data are provided as a Source Data file. All data are presented as mean values. All error bars represent standard error. This immunoblot experiment was repeated twice, with each independent replicate showing similar results.

Supplementary Figure 4.

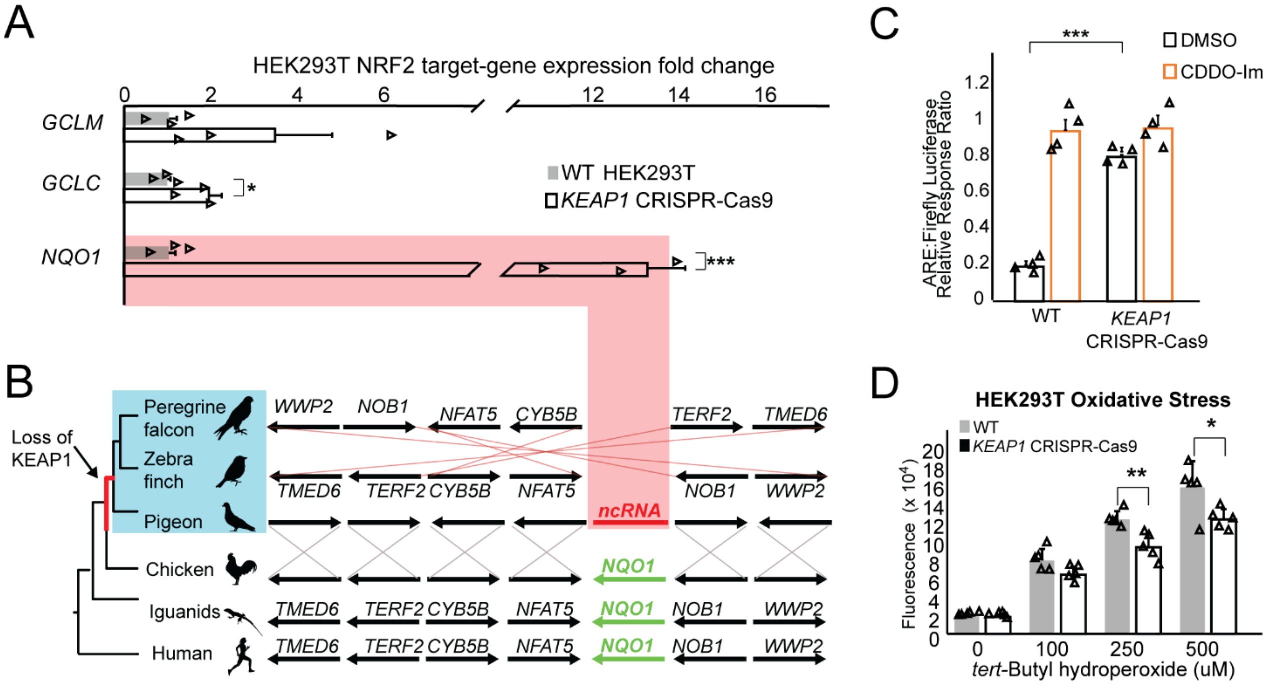

Increased NRF2 activity and decreased oxidative stress in KEAP1-gRNA-Cas9 treated HEK293T cells. (**A**) Disruption of KEAP1 function through CRISPR-Cas9 genome editing in HEK293T cells leads to a significant overexpression of *NQO1*—an important NRF2-regulated detoxification enzyme – and *GCLC* [32] [*N*=3; one-sided t-test]. (**B**) *NQO1* is deleted in Neoaves genomes despite high syntenic conservation of neighboring genes across Human, Anolis, and Chicken. (**C**) When treated with 50nM of the KEAP1-mediated NRF2-activator CDDO-Im [66], the induced NRF2(ARE)-driven Firefly luciferase expression in Cas9-RNP-treated HEK293T cells is significantly muted relative to that of WT [*N*=4 biologically independent cells; p=5.6 $\times 10^{-7}$, one-sided t-test]. (**D**) Decreased cellular oxidative stress results from KEAP1 loss-of-function in HEK293T cells treated with *tert*-Butyl hydroperoxide [*N*=5 biologically independent cells; 250uM tBh, p=0.002; 500uM tBh, p=0.017; one-sided t-test]. Source data are provided as a Source Data file. All data are presented as mean values. All error bars represent standard error.

Supplementary Figure 5.

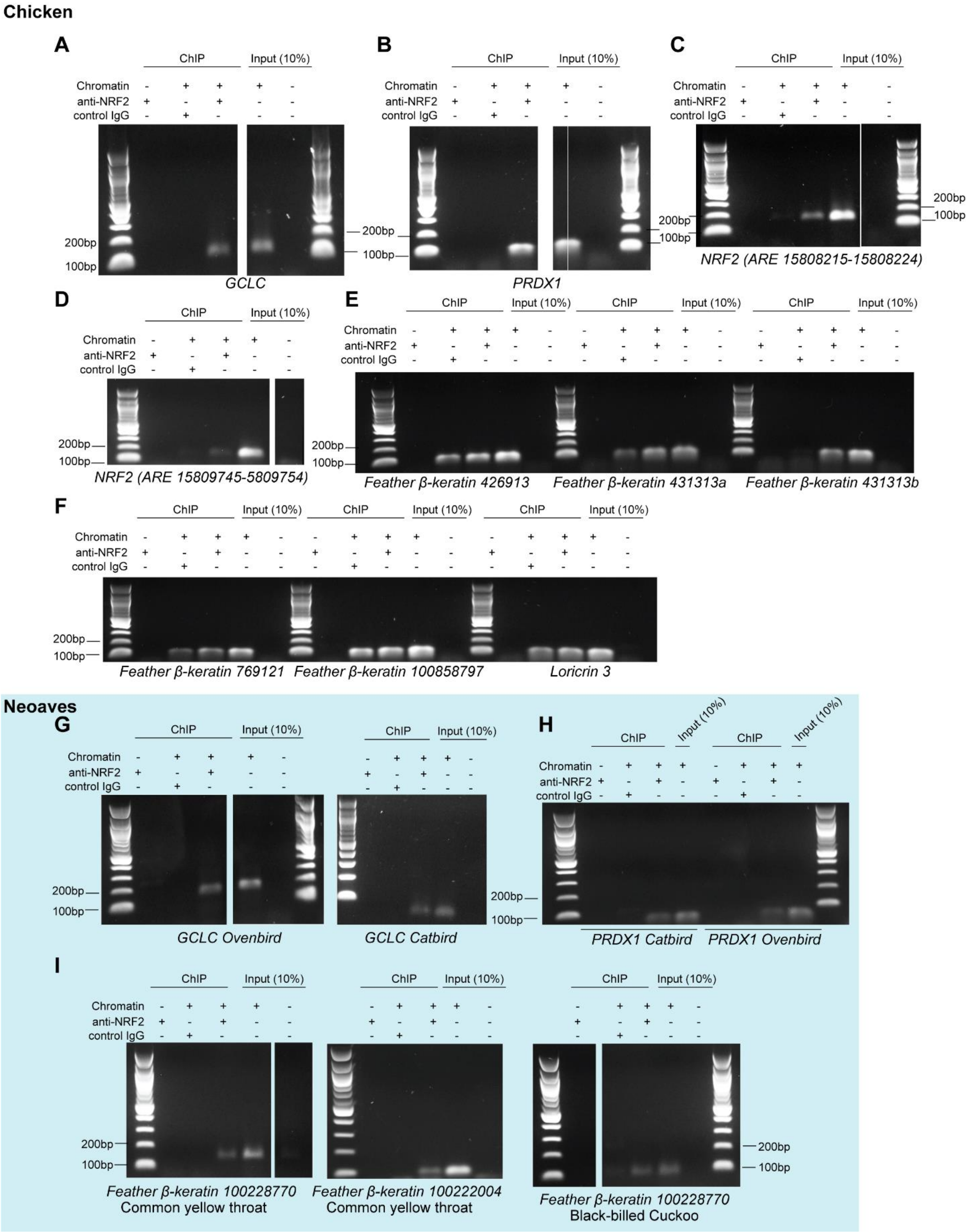

Validation of PCR amplicons quantified in qPCR analysis of Avian Chromatin. These experiments were repeated twice each, with each independent replicate showing similar results. Source data are provided as a Source Data file.

Supplementary Figure 6.

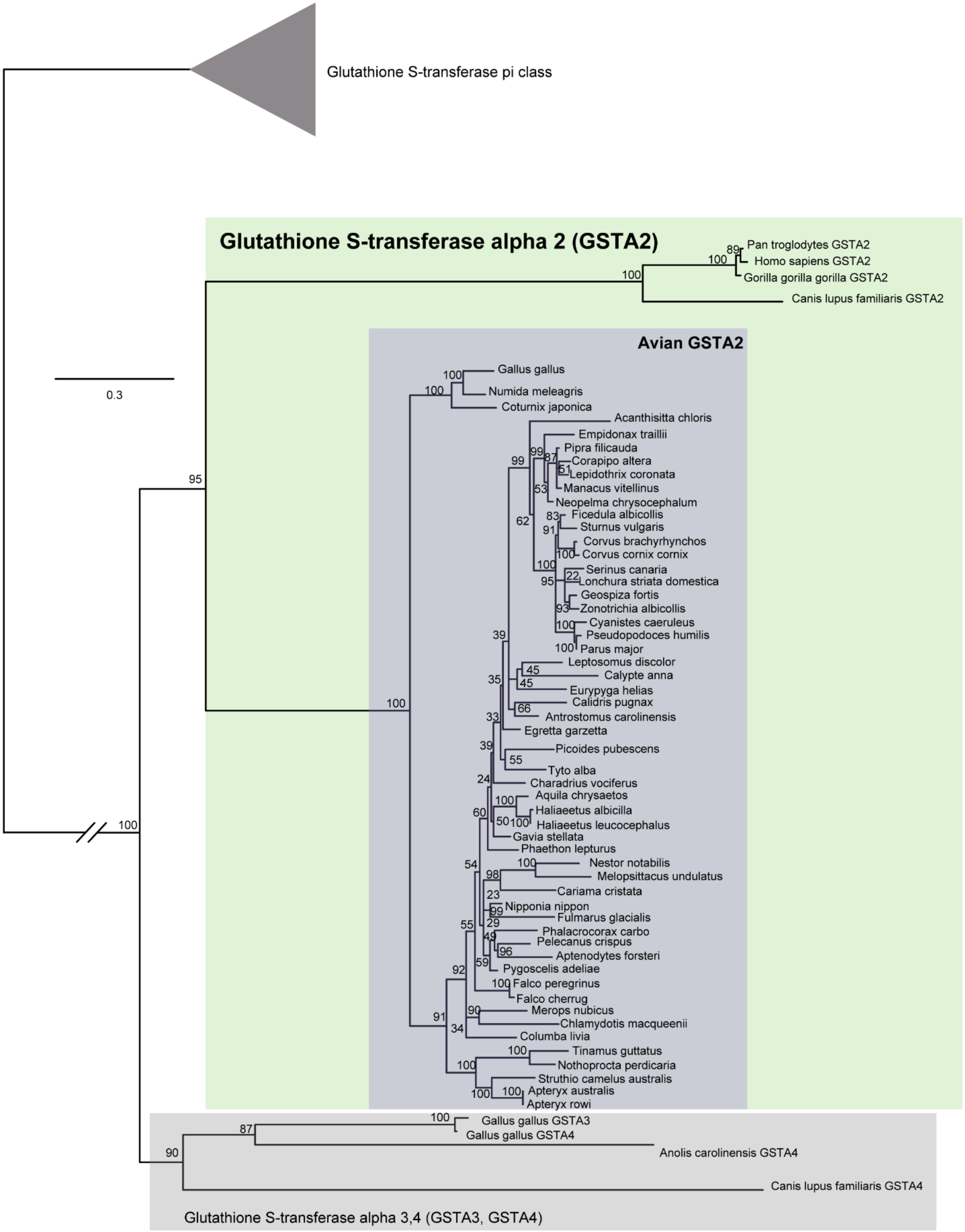

GSTA2 gene tree generated to confirm identity of GSTA2 coding-sequences

Supplementary Figure 7.

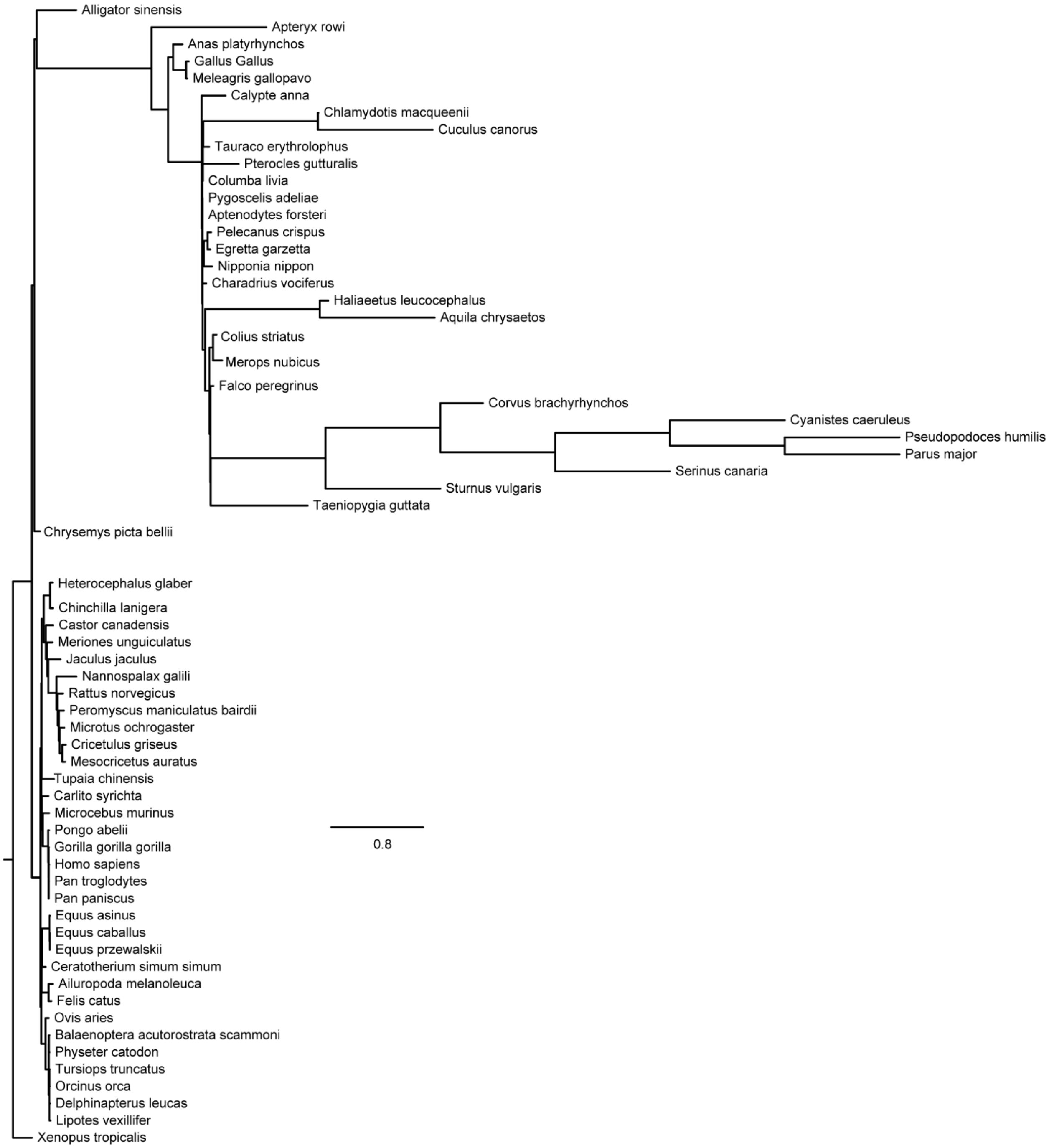

Species tree used in statistical analyses of *KEAP1* evolutionary rates, with branch lengths estimated by PAML.

Supplementary Figure 8.

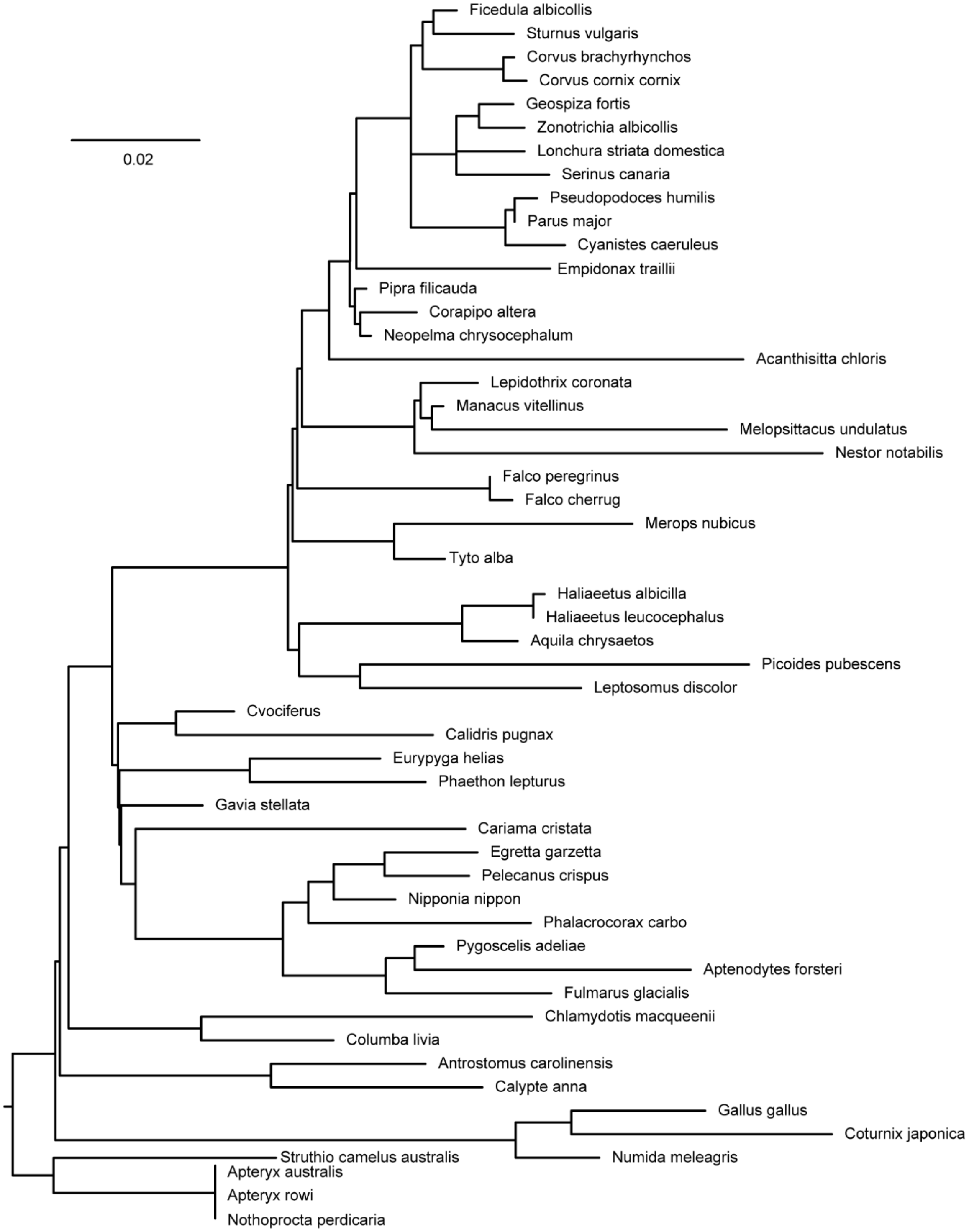

Species tree used in statistical analyses of *GSTA2* evolutionary rates, with branch lengths estimated by PAML.

Supplementary Figure 9

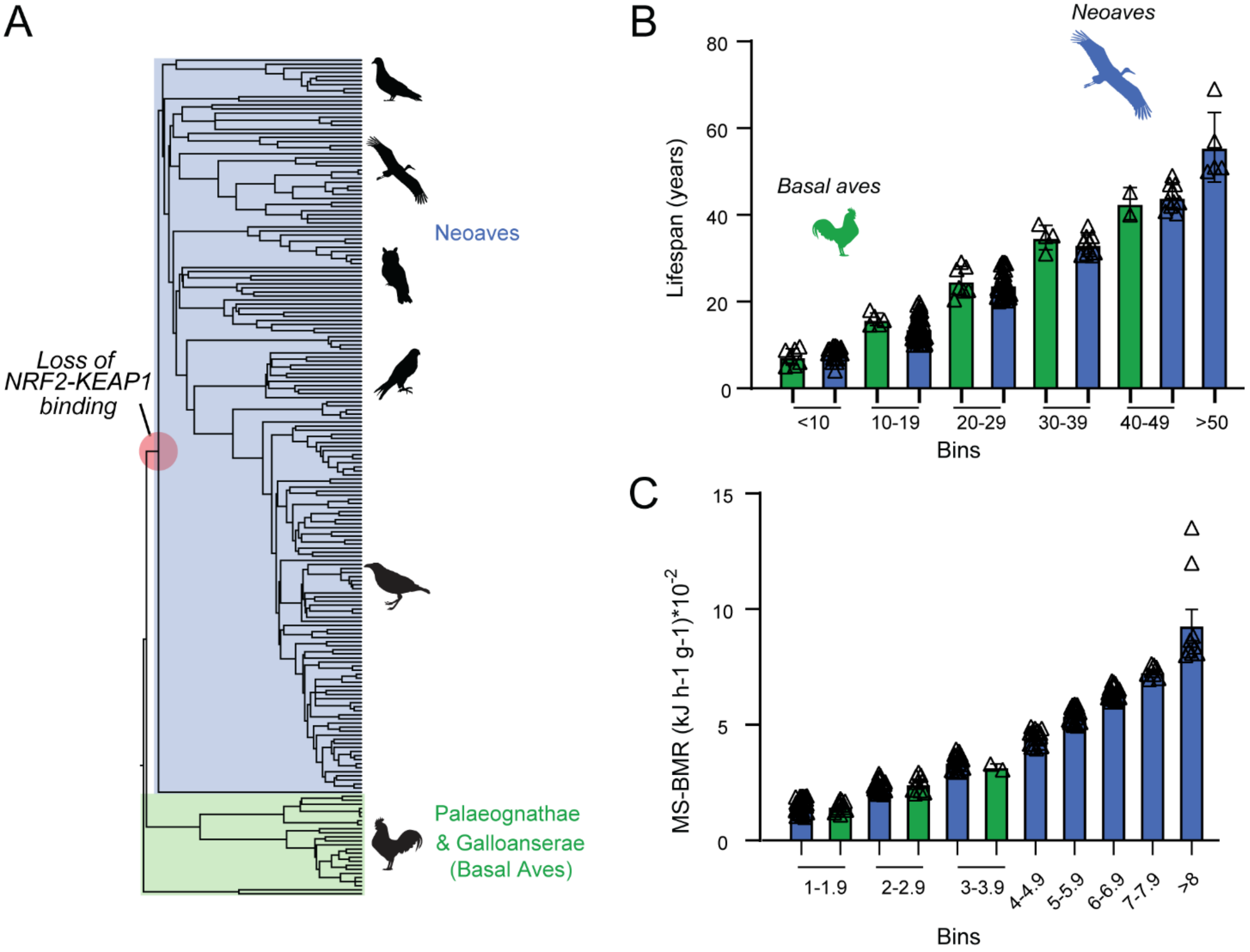

(**A**) Phylogenetic relationships of avian taxa for which both MS-BMR and lifespan data were available (Methods). Red highlight denotes the Neoavian ancestor where KEAP1 binding of NRF2 was lost. Neoaves and basal Aves species stratified into (**B**) lifespan groups [Neoaves: n=180 biologically independent animals; Basal Aves: n=25 biologically independent animals] and (**C**) mass-specific basal metabolic rate (MS-BMR) groups [Neoaves: n=175 biologically independent animals; Basal Aves: n=23 biologically independent animals]. Within each group the mean lifespans and MS-BMR of basal Aves and Neoaves were statistically indistinguishable (Mann-Whitney; Supplementary Table 11). All error bars represent standard error. Source data are provided as a Source Data file. All data are presented as mean values.

Supplementary Table 1.
Non-avian genome accession numbers

scientific name	accession
Drosophila melanogaster	NP_788685.1
Danio rerio	XM_021476739
Latimeria chalumnae	XM_005994311
Xenopus tropicalis	NM_001008023
Lipotes vexillifer	XM_007460670
Delphinapterus leucas	XM_022557191.1
Orcinus orca	XM_012535762
Tursiops truncatus	XM_019933070
Physeter catodon	XM_024134256
Balaenoptera acutorostrata scammoni	XM_007168981
Ovis aries	XM_027969637
Felis catus	XM_023245303
Ailuropoda melanoleuca	XM_002921371
Equus przewalskii	XM_008537450.1
Equus caballus	XM_023645423
Equus asinus	XM_014865526
Ceratotherium simum simum	XM_004442630
Pan paniscus	XM_008961848
Pan troglodytes	XM_009434584
Homo sapiens	XM_011528452.1
Gorilla gorilla gorilla	XM_019015776.1
Pongo abelii	XM_009252776
Microcebus murinus	XM_012790647
Carlito syrichta	XM_008050920
Mesocricetus auratus	XM_021232069
Cricetulus griseus	XM_027411505
Microtus ochrogaster	XM_013346291
Peromyscus maniculatus bairdii	XM_006987015
Rattus norvegicus	XM_006242591
Nannospalax galili	XM_008850040
Jaculus jaculus	XM_004667452
Meriones unguiculatus	XM_021649200
Castor canadensis	XM_020174298
Heterocephalus glaber	XM_004865503
Chinchilla lanigera	XM_013505131
Tupaia chinensis	XM_027765540
Python bivittatus	XM_007426568
Pogona vitticeps	XM_020798692
Anolis carolinensis	XM_003216399
Chrysemys picta bellii	XM_008175416

Alligator sinensis	XM_006023442.3
Ctenopharyngodon idella (Keap1b)	KJ729125
Cyprinus carpio (Keap1a)	JX470752
Danio rerio (Keap1a)	NM_182864

Supplementary Table 2.

Analyses of selection on Mammalian KEAP1 using PAML random sites models.

**Model**	***ln*L**	**Parameters**[1]			**Null**	***p* [df]**[2]
		ω_0**/p**	ω_1**/q**	ω_2/ω_p		
M0	-10727.53	0.01	-	-	N/A	-
M1a	-10699.67	0.01 (98%)	1.00 (2%)	-	M0	1 [1]
M2a	-10699.67	0.01 (98%)	1.00 (1%)	1.00 (1%)	M1a	1 [2]
M3	-10565.06	0.00 (68%)	0.03 (26%)	0.18 (6%)	M0	**0.000** [4]
M7	-10569.21	0.10	4.24	-	N/A	-
**M8**	-10569.21	0.10	4.24	1.00	M7	1 [2]

[1] ω values of each site class are shown are shown for model M0-M3 (ω_0– ω_2) with the proportion of each site class in parentheses. For M7 and M8, the shape parameters, p and q, which describe the beta distribution are listed instead. In addition, the ω value for the positively selected site class (ω_p, with the proportion of sites in parentheses) is shown for M8.

[2] Significant *p*-values ($\alpha \leq 0.05$) are bolded. Degrees of freedom are given in square brackets after the *p*-values. Significance was determined through a likelihood-ratio test of null and alternative models, with reference to a χ^2 distribution.

Abbreviations—***ln*L**, ln Likelihood; ***p***, *p*-value; **N/A**, not applicable.

Supplementary Table 3.

Analyses of recombination (GARD HYPHY) in Avian KEAP1 coding-sequences.

**Breakpoints**	**AICc**	**Delta AICc**	**Segments** (human nucleotide numbering)	**Significant topological incongruence breakpoints (**LHS; RHS p-values**)**[1]
0	53827.95	----	166-1896	----
1	50358.33	3469.62	166-1430; 1431-1896	----
2	48310.18	2048.15	166-630; 631-1411; 1412-1896	631 (0.0004;0.0004); 1412 (0.0004;0.0004)

[1] Shimodaira and Hasegawa (1999) test (SH test) as described in Ref 73.

Supplementary Table 4.
Accession numbers for sequences generated in this study

Definition	Accession
Gallus gallus NF-E2-related factor 2 (NRF2) mRNA complete cds	MN416129
Zonotrichia albicollis Kelch-like ECH-associated protein 1 (KEAP1) mRNA partial cds	MN416130
Zonotrichia albicollis NF-E2-related factor 2 (NRF2) mRNA complete cds	MN416131
Gallus gallus Kelch-like ECH-associated protein 1 (KEAP1 mRNA complete cds	MN416132
Zonotrichia albicollis Kelch-like ECH-associated protein 1 (KEAP1) gene, partial cds	MN416133

Supplementary Table 5.

Results of Clade Model D (CmD) analyses of Tetrapod *KEAP1* under various partitions.

**Model & Foreground**[1]	***ΔAIC***	***ln*L**	**Parameters**[3]			**Null**	***p* [df]**[4]
			ω_0	ω_1	ω_2/ω_d		
M3	10.25	-11019.64	0.001(38%)	0.02(48%)	0.13 (14%)	N/A	-
CmD_*Passerines*	7.44	-11017.24	0.002 (41%)	0.12 (16%)	0.01 (44%) Passerines:0 .04	M3	**0.02** [1]
CmD_*Birds vs. Mammals*	**0.00***	-11012.51	0.001 (41%)	0.02 (46%)	0.25 (13%) Mammals: 0.08 Birds: 0.17	M3	**0.000** [2]

[1]The foreground partition is listed after the underscore for the clade models and consists of either: the clade of 'Passerines', 'Mammals', or the clade 'Birds'. In any partitioning scheme, the entire clade was tested, and all non-foreground data are present in the background partition.
[2]All ΔAIC values are calculated from the lowest AIC model. The best fits are bolded with an asterisk (*).
[3]ω_d is the divergent site class, which has a separate value for the foreground and background partitions.
[4]Significant *p*-values ($\alpha \leq 0.05$) are bolded. Degrees of freedom are given in square brackets after the *p*-values. Significance was determined through a likelihood-ratio test of null and alternative models, with reference to a χ^2 distribution.
Abbreviations—***ln*L**, ln Likelihood; ***p***, *p*-value; **AIC**, Akaike information criterion

Supplementary Table 6.

Avian specimens collected and analyzed in this study

Experiment	Common name (biological replicates)	Species	Collection date
qPCR	American Woodcock (n=2)	*Scolopax minor*	October 2018
	White-throated sparrow (n=3)	*Zonotrichia albicollis*	
ChIP, IB	Ovenbird (n=4)	*Seiurus aurocapilla*	May 2019
	Black-billed cuckoo (n=1)	*Coccyzus erythropthalmus*	
ChIP	Common yellowthroat (n=2)	*Geothlypis trichas*	
ChIP, IF	Gray catbird (n=1)	*Dumetella carolinensis*	
ChIP, IB, IF qPCR	Chicken (n=5)	*Gallus gallus*	Dec 2018/May 2019

ChIP= chromatin immunoprecipitation; IB= immunoblot; qPCR= quantitative PCR; IF= immunofluorescence

Supplementary Table 7.
Antioxidant response elements (ARE) detected in avian genomes

Reference Organism	Genome (Chromosome accession)	Gene	ARE	Coordinates	
**Chicken**	Chicken GRCg6a (Chr 3 NC_006090.5)	GCLC	TGACACAGCT	88173981	88173990
	Chicken GRCg6a (Chr 8 NC_006095.5)	PRDX1	TGAGAAAGCC	21508092	21508101
	Chicken GRCg6a (Chr7 NC_006094.5)	NFE2L2	TGAGACAGCA	15809745	15809754
			TGAGAAAGCG	15808215	15808224
	Chicken GRCg6a (Chr25 NC_006112.4)	Feather keratin 1-like	TGAGTAAGCA	2178857	2178866
		feather beta keratin-like	TGACAAATCT	2270450	2270459
			TGAGACATCT	2270135	2270144
		feather keratin 1-like	TGAGAAAGCA	3122498	3122507
		feather keratin 1-like	TGAGTCATCG	3125460	3125469
		Loricrin 3	TGACACAGCC	3224257	3224266
	Chicken GRCg6a (Chr 3 NC_006090.5)	GSTA2	TGACAAAGCT	88412260	88412269
Zebra finch	Taeniopygia_guttata 3.2.4 (Chr 3 NC_011466.1)	GCLC	TGACACAGCT	90519291	90519300
	Taeniopygia_guttata 3.2.4 (GCF_000151805.1) NC_011471.1	NFE2L2	None	N/A	N/A
Common Cuckoo	Cuculus canorus (GCF_000709325.1) NW_009243908.1	NFE2L2	none	N/A	N/A
Manakin	Manacus vitellinus (GCF_001715985.3) NW_021939471.1	NFE2L2	None	N/A	N/A
Common Canary	Serinus canaria SCA1 (GCF_000534875.1) NW_007931131.1	PRDX1	TGAGTCATCT	17942995	17943004
Starling	Sturnus_vulgaris-1.0(GCF_001447265.1) NW_014650503.1		TGAGTCATCT	1002016	1002025
Sparrow	GeoFor_1.0(GCF_000277835.1) NW_005054347.1		TGAGTCATCT	3368547	3368556
Zebra finch	Zebrafinch 3.2.4 (Chr 25 NC_011489.1)	feather keratin 1-like	TGACAAATCA	789704	789714

		feather keratin 4-like	TGACAAATCA	795758	795767

Supplementary Table 8.

GSTA2 accession numbers (**presence of ARE investigated**)

Name	accession
Apteryx australis	XM_013953262
Acanthisitta chloris	XM_009084876
Aquila chrysaetos	XM_030037898
Aptenodytes forsteri	XM_009288570
Apteryx rowi	XM_026086143
Corapipo altera	XM_027641169
Calypte anna	XM_008495629
Corvus brachyrhynchos	XM_008637649
Cyanistes caeruleus	XM_023922506
Antrostomus carolinensis	XM_010170651
Corvus cornix cornix	XM_010406318
Cariama cristata	XM_009709554
Coturnix japonica	XM_015859291
Columba livia	XM_005506790
Chlamydotis macqueenii	XM_010116437
Calidris pugnax	XM_014948928
Charadrius vociferus	XM_009882144
Egretta garzetta	XM_009638768
Eurypyga helias	XM_010160429
Empidonax traillii	XM_027898226
Ficedula albicollis	XM_005044193
Falco cherrug	XM_027809110
Fulmarus glacialis	XM_009585672
Falco peregrinus	XM_027787880
Gallus gallus	XM_015284825
Geospiza fortis	XM_005426381
Gavia stellata	XM_009812395
Haliaeetus albicilla	XM_009922637
Haliaeetus leucocephalus	XM_010561073
Lepidothrix coronata	XM_017814538
Leptosomus discolor	XM_009956935.1
Lonchura striata domestica	XM_021551287
Merops nubicus	XM_008940286
Melopsittacus undulatus	XM_005153205
Manacus vitellinus	XM_018088908
Neopelma chrysocephalum	XM_027690338
Numida meleagris	XM_021389848
Nipponia nippon	XM_009461358
Nestor notabilis	XM_010022308
Nothoprocta perdicaria	XM_026034283
Pygoscelis adeliae	XM_009333197
Phalacrocorax carbo	XM_009508114

Pelecanus crispus	XM_009478080
Pipra filicauda	XM_027715482
Pseudopodoces humilis	XM_005521908
Phaethon lepturus	XM_010296442
Parus major	XM_015623193
Picoides pubescens	XM_009904173
Struthio camelus australis	XM_009677028
Serinus canaria	XM_018920082
Sturnus vulgaris	XM_014869515
Tyto alba	XM_009962792
Zonotrichia albicollis	XM_005495970

Supplementary Table 9.

Analyses of selection on Avian *GSTA2* using PAML random sites models.

**Model**	***ln*L**	**Parameters**[1]			**Null**	***p* [df]**[2]	**Δ AIC**[3]
		**ω_0/p**	**ω_1/q**	**ω_2/ω_p**			
M0	-6143.41	0.24	-	-	N/A	-	699.9
M1a	-5844.16	0.05 (79%)	1.00 (21%)	-	M0	**0.000** [1]	103.38
M2a	-5802.86	0.05 (78%)	1.00 (19%)	3.87 (3%)	M1a	1 [2]	24.78
M3	-5796.21	0.03 (73%)	0.68 (24%)	3.51 (3%)	M0	**0.000** [4]	13.49
M8a	-5827.47	0.27	2.58	1.00 (13%)	N/A	**-**	72
M7	-5835.17	0.14	0.49	-	N/A	-	85.4
**M8**	-5790.47	0.17	0.71	3.49 (2%)	M7	**0.000** [2]	**0***
					M8a	**0.000 [**1]	

[1]ω values of each site class are shown are shown for model M0-M3 (ω_0– ω_2) with the proportion of each site class in parentheses. For M7 and M8, the shape parameters, p and q, which describe the beta distribution are listed instead. In addition, the ω value for the positively selected site class (ω_p, with the proportion of sites in parentheses) is shown for M8.
[2]Significant *p*-values ($\alpha \leq 0.05$) are bolded. Degrees of freedom are given in square brackets after the *p*-values. Significance was determined through a likelihood-ratio test of null and alternative models, with reference to a χ^2 distribution.
[3] Model fits were assessed by Akaike information criterion differences to the best fitting model (bolded asterisk).
Abbreviations—***ln*L**, ln Likelihood; ***p***, *p*-value; **N/A**, not applicable.

Supplementary Table 10.

Conventional and phylogenetically-independent non-parametric Kruskal-Wallis tests comparing mass-specific basal metabolic rate (MS-BMR) or lifespan of various groups using non-functional (0) vs functional (1) KEAP1-based categories

**Group[1]**	**Category**	**Response Variables**	**N**	**Median**	**Mean Rank**	**Z-value**	**DF**	**H-value**	**Conventional P value**	**Phylogenetic P value***
<10 Lifespan	0	MS-BMR	7	0.021	4.7	-3.81	1	14.53	**0.000**	**0.03**
	1		27	0.061	20.8	3.81				
10-19.9 Lifespan	0		5	0.017	14.0	-2.80	1	7.83	**0.005**	**0.02**
	1		87	0.049	48.4	2.80				
Neoaves >0.08, basal Aves 0.02-0.029, MS-BMR	0	Lifespan	9	10.1	8.1	-1.10	1	1.22	0.270	---
	1		9	14.7	10.9	1.10				

Degrees of freedom (DF)

[1]Data was grouped according to either lifespan (years) or MS-BMR (kJ/h/g; see figures 5C-D, respectively).

*Only significant conventional P values (<0.05) were further investigated for phylogenetic significance. Phylogenetic significance was determined by empirical null distribution generated by phylogenetic simulations. Bold P values are significant.

Supplementary Table 11.

Conventional Mann-Whitney testing equivalency between basal Aves vs Neoaves MS-BMR and lifespan stratifications

Group[1]	Sample	N	Median	W-value	P value
<10 Lifespan	Basal Aves	7	7.4	99.0	0.33
	Neoaves	27	8.6		
10-19 Lifespan	Basal Aves	5	15.7	344.0	0.06
	Neoaves	87	13.1		
20-29 Lifespan	Basal Aves	7	23.2	199.5	0.42
	Neoaves	41	22.9		
30-39 Lifespan	Basal Aves	4	35.1	42.0	0.21
	Neoaves	11	33.7		
40-49 Lifespan	Basal Aves	2	42.6	9.0	0.45
	Neoaves	10	43.2		
0.01-0.019 MS-BMR	Basal Aves	12	0.015	256	0.44
	Neoaves	35	0.016		
0.02-0.029 MS-BMR	Basal Aves	9	0.025	203	0.453
	Neoaves	30	0.023		
0.03-0.039 MS-BMR	Basal Aves	2	0.032	18	0.55
	Neoaves	21	0.034		

[1]Data was grouped according to either lifespan (years) or MS-BMR (kJ/h/g ; see figures S9B-C, respectively).

Supplementary Table 12.

Primer sequences used in this study

	**Template Species**	**Gene**	**Forward**	**Reverse**
Avian NRF2 KEAP1 cloning	Chicken	KEAP1	ATGTACGGCCCCGAATGCCA	ACAGGGGCACTCGTGGCG
		NRF2	ATGAACTTGATTGACATCCTTTGGAGGC	CAGTTTAGTCTCTGCCTTTCTGCTTTTA
	White-throated sparrow	NRF2	ATGAACCTGATTGACATCCTTTGGAGG	AAGTTTAGTCCCCGACTGCCTG
		KEAP1	CCAGCCCCGTCTTCAGGGCCATGTTCACAGCGAGGCTGCG	CCCGGTTTGCACGGCTCCATGGTGACGGCCACGCTCCAG
ChIP qPCR	Chicken	GCLC	CTCATGTGGTAACGCCACCAAAG	TGTGTCACAAGGGAAGAGTTCTGC
		PRDX1	TCGGCATTCACCGGGCTTTA	CTTTCTCATGGCGCAGTGCTTA
			CATCTGACTCATCCTGCCGTGA	CTCAGCGCTTGGATGGTGG
		NFE2L2	GTAGTGGTAGTGGCACAGGTATGAAA	GCCACAAGACAGCAACCTGTGA
			GGGACAATTTGGCCCCTGGAAAT	CTACCTCAGCAATGCAGATCCCTTT
		Feather keratin 1-like	GTGCCATGACTGGAAAGGTGATTTT	CAGGCAAGGAGAGCCCACATTAA
		feather beta keratin-like	CTCGTGTCTGAGCTGACAAATCTGG	GAGAGTGAGGAGTGATGTGCAACA
			GCACCCAAGTGGATGAGACATCTC	CTCCTCCTTGGCAAAGACTCCTT
		feather keratin 1-like	CCTTAGCAGGAAGTCTGAGAAAGCA	TCAGATCCCCATGCTGAGGGTATA
		feather keratin 1-like	CAACAGTCATGAGTCATCGTGAGCT	AAGTCACCTTCCCTCCTCTGGTT
		Loricrin 3	CATTGCTTTGACACAGCCACTGT	AGCAGCACCGCTCACACT
	Neoaves species	GCLC	CTCACTGCCCCAAAGCTAGAAAG	TGATAGCCAACAGCTGTGTCAG
		PRDX1	CATCTGGTCCCAAAATGAGTCATCTCT	GTGTAGTGTTGCTGGGTGACCATAC
			TGAGTCATCTCTGGGTGAACC	
		feather keratin 1-like	GGAGGGCCAGATGGTCAGT	GCAGGACGTGCCCAGTCA
		feather keratin 4-like	GGAACAGGAGGGGTTTAGAGGTT	AATTTGAGGCACAGCTGGAAGAAC
HEK Cell qPCR	Human	GCLM	TGTGTGATGCCACCAGATTT	TTCACAATGACCGAATACCG
		GCLC	ACCATCATCAATGGGAAGGA	GCGATAAACTCCCTCATCCA
		HO1	AACTTTCAGAAGGGCCAGGT	GTAGACAGGGGCGAAGACTG
		GSTM1	TTCCCAATCTGCCCTACTTG	TCTCCAAAATGTCCACACGA
		NQO1	CAGCTCACCGAGAGCCTAGT	ACCACCTCCCATCCTTTCTT
Avian qPCR	Chicken and	NRF2	GCAGAACATAGAGCAAGTTTGGGAAG	GTGCTCACCTCAGCCAGGTT

	Neoaves species	GAPDH	GGAGGGTCTCATGACCACTGTC	CAGGAGACAACCTGGTCCTCT
		GCLC	CTAGAGCCACTGAAGAACAACC	GTGTGCAATATGCTGTGCCAA
		GCLM	CARDAAGATTGTTGCCATAGG	CATCACRCAACAKGAAGCTAG
		PRDX1	TGCATACAGRGGTCTSTTCA	CTTCCAGCCAGCTGGGCACA
		TXNRD1	GRTTTCMCATTGCCACTGGTGAA	TARGAAGCTCCAACCACCAG
		PTGR1	GGAGAGTTGCTGCTTGAATCAGTGTTTC	GCAACCTGCGTGCCAATCATTA
			GAGAGCTGCTGCTTGAATCAGTGTTT	GCAACCTGCGTGCCTATCATTATG
T7EI assay	Human	KEAP1	GGCTAATTGCGTAGGGCTTTGCA	TCAATGAGGCGCTCCATGACC
		TNXB	CCACTCTTCCTAGTGCCTCAGAGT	CCAAGTCCCGCTCACAGGAT
		LOC105372218	TGCTGGAGGGAGAGGCAC	ACAGCGTGCACAGACCCA

Supplementary Table 13.

crRNA (Hs.Cas9.KEAP1.1.AD) used in CRISPR-Cas9 genome editing of *KEAP1* in HEK293T cells (information provided by Integrated DNA Technologies)

Position	Strand	Sequence	PAM	On-Target Score	Off-Target Score (Genes and loci)
10499768	-	GGTCAAGTACCAGGATGCAC	CGG	98	71 (TNXB chr6:-32081514;LOC105372218 chr18:-77983723)
