# Supplementary material for: Adaptation of the master antioxidant response connects metabolism, lifespan and feather development pathways in birds
Source: Nat Commun. 2020 May 18;11:2476. doi: 10.1038/s41467-020-16129-4 (PMC7234996; doi:10.1038/s41467-020-16129-4)
Supplement: Supplementary file 3 — Reporting Summary [file 41467_2020_16129_MOESM3_ESM.pdf]

nature research

Corresponding author(s): Elia Duh

Last updated by author(s): Apr 2, 2020

Reporting Summary

Nature Research wishes to improve the reproducibility of the work that we publish. This form provides structure for consistency and transparency in reporting. For further information on Nature Research policies, see Authors & Referees and the Editorial Policy Checklist .

Statistics

For all statistical analyses, confirm that the following items are present in the figure legend, table legend, main text, or Methods section.

n/a	Confirmed	
☐	☒	The exact sample size (n) for each experimental group/condition, given as a discrete number and unit of measurement
☐	☒	A statement on whether measurements were taken from distinct samples or whether the same sample was measured repeatedly
☐	☒	The statistical test(s) used AND whether they are one- or two-sided<br>*Only common tests should be described solely by name; describe more complex techniques in the Methods section.*
☒	☐	A description of all covariates tested
☐	☒	A description of any assumptions or corrections, such as tests of normality and adjustment for multiple comparisons
☐	☒	A full description of the statistical parameters including central tendency (e.g. means) or other basic estimates (e.g. regression coefficient) AND variation (e.g. standard deviation) or associated estimates of uncertainty (e.g. confidence intervals)
☐	☒	For null hypothesis testing, the test statistic (e.g. F, t, r) with confidence intervals, effect sizes, degrees of freedom and P value noted<br>*Give P values as exact values whenever suitable.*
☐	☒	For Bayesian analysis, information on the choice of priors and Markov chain Monte Carlo settings
☒	☐	For hierarchical and complex designs, identification of the appropriate level for tests and full reporting of outcomes
☒	☐	Estimates of effect sizes (e.g. Cohen's d, Pearson's r), indicating how they were calculated

Our web collection on statistics for biologists contains articles on many of the points above.

Software and code

Policy information about availability of computer code

Data collection	All public genomic data was obtained from NCBI. No software was used for data collection.
Data analysis	IQ-Tree Web Server, UCSF Chimera package (v1.13.1) , PAML v 4.7, Minitab 19, MEGA 10, HYPHY datamonkey web server, ImageJ (v1.52d), Procheck (v3.5), ProSA-web

For manuscripts utilizing custom algorithms or software that are central to the research but not yet described in published literature, software must be made available to editors/reviewers. We strongly encourage code deposition in a community repository (e.g. GitHub). See the Nature Research guidelines for submitting code & software for further information.

Data

Policy information about availability of data

All manuscripts must include a data availability statement. This statement should provide the following information, where applicable:

- Accession codes, unique identifiers, or web links for publicly available datasets
- A list of figures that have associated raw data
- A description of any restrictions on data availability

NCBI accession numbers generated by this study are listed in Supplementary Table 4 (MN416129 [https://www.ncbi.nlm.nih.gov/nuccore/MN416129], MN416130 [https://www.ncbi.nlm.nih.gov/nuccore/MN416130], MN416131 [https://www.ncbi.nlm.nih.gov/nuccore/MN416131], MN416132 [https://www.ncbi.nlm.nih.gov/nuccore/MN416132], MN416133 [https://www.ncbi.nlm.nih.gov/nuccore/MN416133]). All other data are available in the main text and Supplementary Materials. Figures with raw data are described in the source data file.

Field-specific reporting

Please select the one below that is the best fit for your research. If you are not sure, read the appropriate sections before making your selection.

- [x] Life sciences
- [ ] Behavioural & social sciences
- [ ] Ecological, evolutionary & environmental sciences

For a reference copy of the document with all sections, see nature.com/documents/nr-reporting-summary-flat.pdf

Life sciences study design

All studies must disclose on these points even when the disclosure is negative.

Sample size	At least three biological replicates were used for all statistical analyses involving molecular biology (qPCR, western blot) and cellular assays (luciferase, oxidative stress, immunocytochemistry). DNA consensus sequences were derived from >5 technical replicates. Areas where available avian individuals of the same species were less than three is clearly specified in the figure captions and main text. Sample sizes were determined by availability of specimens.
Data exclusions	No data were excluded from the analyses
Replication	We replicated gene and protein expression level patterns across multiple species, as a control for phylogenetic divergence. Experiments were replicated at least twice, and all attempts at replication were successful.
Randomization	Organisms were grouped based off species-specific genetic mutations
Blinding	Blinding was not relevant due to prior knowledge of experimental groups that had been based off genetic analysis

Reporting for specific materials, systems and methods

We require information from authors about some types of materials, experimental systems and methods used in many studies. Here, indicate whether each material, system or method listed is relevant to your study. If you are not sure if a list item applies to your research, read the appropriate section before selecting a response.

Materials & experimental systems

n/a	Involved in the study	
☐	☒	Antibodies
☐	☒	Eukaryotic cell lines
☒	☐	Palaeontology
☐	☒	Animals and other organisms
☒	☐	Human research participants
☒	☐	Clinical data

Methods

n/a	Involved in the study	
☒	☐	ChIP-seq
☒	☐	Flow cytometry
☒	☐	MRI-based neuroimaging

Antibodies

Antibodies used	anti-HA (C29F4 Cell Signaling Technology), anti-Myc (71D10 Cell Signaling Technology), anti-beta actin (13E5 Cell Signaling Technology) anti-NRF2 (16396-1-AP, Proteintech Group), anti-KEAP1 (D6B12 Cell Signaling Technology), and anti-Lamin B1 (D4Q4Z Cell Signaling Technology), anti-β-tubulin (9F3 Cell Signaling Technology) and anti-Histone H3 (D1H2 Cell Signaling Technology). anti-rabbit IgG conjugated with Alexa fluor 594 (Invitrogen), anti-rabbit IgG conjugated with Alexa fluor 594 (Invitrogen), rat IgG conjugated with Alexa fluor 488 (Invitrogen)
Validation	All antibodies were verified by western blot analysis of Human cell lysates (HEK293T)

Eukaryotic cell lines

Policy information about cell lines

Cell line source(s)	ATCC (HEK293T), Charles River (chicken primary fibroblasts), wild Dumetella carolinensis (Neoaves primary fibroblasts)
Authentication	ATCC HEK293T cells were authenticated by ATCC STR profiling prior to purchase. Primary fibroblasts were derived first-hand from wild specimens, or derived from captive animals by commercial sources . No cell lines were further authenticated after purchase or first-hand derivation.
Mycoplasma contamination	All cell lines were confirmed negative

Commonly misidentified lines (See ICLAC register)	No commonly misidentified cell lines were used in this study.

Animals and other organisms

Policy information about studies involving animals; ARRIVE guidelines recommended for reporting animal research

Laboratory animals	The study did not involve laboratory animals
Wild animals	The study did not involve live wild animals
Field-collected samples	Neoaves individuals killed by window strike conditions were collected by the Baltimore Bird Club chapter of the Maryland Ornithological Society under a US Fish and Wildlife Salvage Permit (MB-197741-0) during October 2018 and May 2019. Species identifications for birds investigated in this study were provided by the Baltimore Bird Club and are described in Table S7. Liver, skin, and muscle samples were dissected from Neoaves specimens under Biosafety Level 2 conditions following Johns Hopkins Biosafety protocols. Chicken tissues were collected immediately after slaughter from a local farm (Locust 499 Point Farms, Elkton MD).
Ethics oversight	Collected bird samples were under a US Fish and Wildlife Salvage Permit (MB-197741-0)

Note that full information on the approval of the study protocol must also be provided in the manuscript.
