# Supplementary material for: Adaptation of the master antioxidant response connects metabolism, lifespan and feather development pathways in birds
Source: Nat Commun. 2020 May 18;11:2476. doi: 10.1038/s41467-020-16129-4 (PMC7234996; doi:10.1038/s41467-020-16129-4)
Supplement: Supplementary file 4 — Description of Additional Supplementary Files [file 41467_2020_16129_MOESM4_ESM.pdf]

Description of Additional Supplementary Files

File Name: Supplementary Data 1

Description: Avian genomes investigated for KEAP1 coding-sequences
