# Supplementary material for: Adaptation of the master antioxidant response connects metabolism, lifespan and feather development pathways in birds
Source: Nat Commun. 2020 May 18;11:2476. doi: 10.1038/s41467-020-16129-4 (PMC7234996; doi:10.1038/s41467-020-16129-4)
Supplement: Supplementary file 2 — Peer Review File [file 41467_2020_16129_MOESM2_ESM.pdf]

Reviewers' comments:

Reviewer #1 (Remarks to the Author):

In this manuscript, the authors found that a mutation in KEAP1 in neoavian birds results in the constitutive activation of NRF2. While this finding is very interesting, there is currently not sufficient experimental support for the physiological function of activated NRF2 signaling in neoaves for publication in Nature Communications.

Major comments

1. The fact that KEAP1 is genetically inactivated in neoaves, resulting in the constitutive activation of NRF2, is very interesting. However, the data related to the physiological consequences of NRF2 activation, in terms of oxidative stress tolerance, plumage, song systems and lifespan, are only correlative, but not causative. To demonstrate that constitutive NRF2 activation is important for lifespan and plumage pathway in birds, loss-of-NRF2-function experiments are required. Even if it is difficult to generate NRF2 knockout birds, loss of NRF2 function experiments using Neoaves primary cells may be possible. Effects of Nrf2 deficiency on expression of its target genes such as GSTA2 and b-keratin should be examined.

2. In Figure 2D, the expression of only four NRF2 target genes is compared. A global analysis of gene expression using a microarray or RNA-seq would be much more informative, and would give a clearer idea of which NRF2-dependent genes are upregulated in neoaves.

3. In Figure 3D, the induction of the luciferase reporter by CDDO-Im in chicken cells is extremely low (only 2-fold). It would be much more informative to check the induction of endogenous gene expression for multiple NRF2 target genes.

4. In Figure 5, when comparing all neoaves with basal aves, the presence of functional KEAP1 was not associated with increased lifespan and basal metabolic rates ($p=0.25$). It does not seem fair to separate the Passeriformes, which have the same KEAP1 mutation.

Minor comments

1. In Figure 1E, NRF2 protein levels should be compared between no treatment and KEAP1 Cas9. The blots should be shown on same membrane.

2. In Figure 1F, are the KEAP1-null HEK293T cells used for the transfection experiments? If so, basal ARE-luciferase activity should be high even in absence of NRF2 transfection.

3. Figure 2B is not cited in text.

Reviewer #2 (Remarks to the Author):

In this manuscript, the authors demonstrated that functional Keap1 is lost at the start of Neoaves evolution and several gene variations coevolved to compensate for the constitutive Nrf2 activation. Furthermore, they demonstrated that the functional loss of Keap1 leads to longer longevity in birds, implying the possible way for human longevity enhancement. This manuscript is well organized, well written and exciting. I have only minor critics.

Minor points:
1. In Figure 3E; the authors should indicate what fluorescence was measured in the figure legend.

2. Line 244 and 303; ARE is an abbreviation of antioxidant response element. Therefore, to say "ARE element" is redundant.
3. Figure 2C; The authors indicated in the figure that "★(star mark) IgG background=undetectable" and indicated the ★(star mark) above each bar except for those for NRF2. This means that IgG background is detected at the NRF2 locus. However, the authors did not describe how they treated it to calculate the results. Furthermore, the mark above the PRDX1 looks like "asterisk" rather than the star mark.
4. Figure 2A; "Fold change NRF2 RNA expression" should read "Fold change NRF2 mRNA expression".
5. Throughout the manuscript; Abbreviations should be fully spelled out only at their appearances.

Reviewer #3 (Remarks to the Author):

General comments:
This is a thoroughly conducted study presenting exciting results of interest to a broad audience. The authors set out to study why birds seem to have superb abilities to counteract oxidative stress (and live comparably long lives) despite excess ROS production caused by their ability to fly and high metabolic rates. The authors target their studies on a main transcription factor (NRF2) in the major cellular pathway that regulates the antioxidant response to intracellular oxidative stress. They find an astonishing difference between Aves (the basal bird groups) and Neoaves (makes up 95% of all bird species). In Aves the NRF2 gene is strongly regulated by a functional KEAP1 gene only allowing the NRF2 to trigger intra-cellular antioxidant defense during stressful conditions. In contrast, in Neoaves the KEAP1 gene is non-functional and the NRF2 gene always expressed resulting in a constitutive intra-cellular antioxidant defense, and they also show that in Neoaves NRF2 proteins are located (constitutively!) in the cell nucleus! This could arguably provide Neoaves with super-efficient antioxidant abilities, which may have facilitated evolution of higher metabolic rates and possibly longer life span. The authors also show that there has been compensatory effects, for example a complete loss of a pro-tumerogenic locus in Neoaves (NQO1) that in Aves (chicken) is upregulated by NRF2, as well as loss of NRF2-binding regulatory sites upstream the GSTA2 gene (identified to be involved in bird song and plumage coloration pathways).
In conclusion, I really liked this study and how it throughout the Results section walked step-by-step to test (and compare between Aves and Neoaves) different aspects of the major antioxidant pathway where NRF2 is a key regulating factor. Super nice and interesting!
Still, I think there are some places where results have been over-interpreted, in particular in the Discussion (see my specific comments below), and the manuscript would become more solid with a careful revision on this point.

Specific comments:
Abstract: Here authors say: 'Our evidence suggests….' '…that this ancient mutation induced a compensatory program in NRF2-target genes associated with feather development and plumage coloration, while enabling significant increases to life span and metabolic rates.'
I agree that the authors have found some evidence for induced compensatory program in one feather development gene (β-keratin) that also may be involved in non-carotenoid plumage coloration. However, the authors talk about 'NRF2-target genes', so implying that they have been analyzing more than one gene. The other gene they investigated was the GSTA2 gene, which was the only gene they found being included in all three gene sets they obtained from the literature search (comparing genes involved in NRF2-regulation in mice, plumage coloration in Neoaves, zebra finch song system). However, I cannot imagine that research experts on bird 'plumage coloration' genes would say that GSTA2 is an important gene in plumage coloration pathways. Most researchers interested in bird plumage coloration would think when they read this Abstract that the authors have investigated key genes involved in carotenoid- and melanin-based plumage colors. There is no such analysis. Revise this to avoid the impression that several genes, both feather development and plumage coloration genes, have been investigated in this study.

I also have some problems with the ending of the sentence in the Abstract quoted above. I am not convinced that the constitutive NRF2 gene activity has had any effect on MS-BMR or maximal life span. The most rigorous test for the purpose of this study is to compare maximal life span between Aves (with KEAP1-regulated NRF2 gene expression) and Neoaves (with constitutive, non-KEAP1 regulated, NRF2 expression) and this test is not significant (p=0.25). And I am not convinced that the follow-up test really shows the claimed significant differences between Aves and Neoaves (see my worries below under Page 15, 3d part).

Page 14, 2nd part, line 7: Here you say that '…these analyses provide in vivo evidence that the evolution of NRF2-associated feather and song system genes has been shaped by the constitutive activation of NRF2 in Neoaves.' However, the only data on any song system related gene is the GSTA2 gene – and as far as I know this is not a key gene in the song system pathway. I suggest you omit 'song system genes' from this sentence. It becomes too much of hand-waiving and over-interpretation of your data – in fact, it takes credit from your meticulous work on the KEAP1 and NRF2 in Aves vs. Neoaves!

Page 15, 3d part, lines 2-9: For the most correct and rigorous test, comparing either life span or MS basal metabolic rate between functional KEAP1 (Aves) vs. non-functional KEAP1 (Neoaves) bird species, no correlations were found (p=0.25). The authors argue that there was a trend for Neoaves to have higher values in both tests, and this may be hinted when examining Figure 5A and 5B by eye. However, no means and SD are presented anywhere in the paper. Moreover, the statistics presentation in Table S11 is impossible to read and understand because it is not possible to connect each response variable (life span or MS-BMR) to the correct row of test information in the Table. The Table S11 needs to be revised for clarity!
Then, the authors divide the KEAP1 factor into three groups and run MANOVA based on this grouping for the two response variables (still impossible to separate in the table). This yields an overall significant result. However, the problem with this is that we do not know if the significant difference is between Aves and the two Neoaves groups, or if the significant difference is within Neoaves (i.e., between Passerines and non-passerine Neoaves). Inspecting the figures by eye suggest to me that the difference is between Passerines and non-passerine Neoaves both for life span and MS-BMR. If so, this would result in a different conclusion than the one given in the present version of the MS.
If you think that Passerines are so different in aspects of size etc., you may in the follow-up analysis exclude Passerines and just compare Aves with non-passerine Neoaves. If in these analyses non-passerine Neoaves shows significantly higher life span and MS-BMR, then you have a case for that constitutive NRF2 activity may increase life span and MS-BMR in non-passerine Neoaves.

Page 16, 2nd part, lines 5-7: Here authors say: '…induced wide-ranging compensatory mutations in NRF2 target-genes associated with plumage coloration and feather development…'. I think it is an over-statement to talk about 'wide-ranging compensatory mutations' when only two genes have been analysed, and only one of these is clearly important in feather development (the gene argued to be involved in plumage coloration (GSTA2) is in fact a gene known to be importantly involved in ROS scavenging). Revise this to avoid over-statement.

Page 16, 2nd part lines 7-8: Here authors write: '…statistically significant increases in avian life span and metabolic rates.' I am not convinced by the statistical analyses used to test this (see my argumentation above under page 15, 3d part). Please reanalyse this.

Page 18 1st part, lines 1-3: See previous point!

Page 19, 2nd part, line8: Instead of citation 53, better cite the original idea paper by von Schantz et al. 1999 ('Good genes, oxidative stress and radical sexual signals' in Proceedings of the Royal Society, B 266:1-12).

Ref: manuscript NCOMMS-19-41140: Adaptation of the master antioxidant response connects metabolism, lifespan and plumage pathways in birds

Reviewer #1:

In this manuscript, the authors found that a mutation in KEAP1 in neoavian birds results in the constitutive activation of NRF2. While this finding is very interesting, there is currently not sufficient experimental support for the physiological function of activated NRF2 signaling in neoaves for publication in Nature Communications.

Response

We thank the reviewer for the positive comments and helpful suggestions and appreciate your time and consideration reviewing our paper.

Major comments

1. The fact that KEAP1 is genetically inactivated in neoaves, resulting in the constitutive activation of NRF2, is very interesting. However, the data related to the physiological consequences of NRF2 activation, in terms of oxidative stress tolerance, plumage, song systems and lifespan, are only correlative, but not causative. To demonstrate that constitutive NRF2 activation is important for lifespan and plumage pathway in birds, loss-of-NRF2-function experiments are required. Even if it is difficult to generate NRF2 knockout birds, loss of NRF2 function experiments using Neoaves primary cells may be possible. Effects of Nrf2 deficiency on expression of its target genes such as GSTA2 and b-keratin should be examined.

Response

We appreciate this feedback and agree that our results do not directly establish causation between the loss of KEAP1 and various avian phenotypes. Indeed, the experiments suggested by the reviewer are of active interest to us. However, this will require very extensive experimentation, and we believe that it is beyond the scope of the current study. We do show strong evidence for a causal role of NRF2 activation in oxidative stress tolerance, and we also provide empirical evidence for the role of NRF2 in the regulation of feather β-keratin genes. Although our lifespan and metabolism analysis is correlative, our results provide an important first step in identifying the potential phenotypic consequences of constitutive NRF2 activity. We have revised the text to more explicitly mention the limitations of our study, with an emphasis on the need for experimental demonstration of the causative role of NRF2 hyperactivity on Neoavian physiology (Lines 412-415; 476-478)**.**

2. In Figure 2D, the expression of only four NRF2 target genes is compared. A global analysis of gene expression using a microarray or RNA-seq would be much more informative, and would give a clearer idea of which NRF2-dependent genes are upregulated in neoaves.

Response

We thank the Reviewer for this valuable feedback. A global analysis of gene expression in Neoaves is indeed of active interest to us. However, our goal in the present study was simply to establish that NRF2 is transcriptionally active in Neoaves tissues and cells. Our results in Figure 2D, as well as in complementary in vitro studies (see below) help demonstrate the constitutively high expression of multiple NRF2 target genes in Neoaves tissues and cells (described below), consistent with the upregulation and nuclear localization of Nrf2 protein in Neoaves.

3. In Figure 3D, the induction of the luciferase reporter by CDDO-Im in chicken cells is extremely low (only 2-fold). It would be much more informative to check the induction of endogenous gene expression for multiple NRF2 target genes.

Response

We thank the reviewer for this very helpful suggestion. We have conducted this experiment, which is presented in the revised Figure 3E (lines 1100-1127) and described in the main text (lines 245-262). The new results clearly show that the endogenous expression of multiple NRF2 target genes are significantly upregulated in chicken cells upon CDDO-Im treatment. Some of these target genes (e.g. PRDX1) are upregulated nearly five-fold relative to control vehicle treatment. In Neoaves cells by contrast, these same target genes are constitutively expressed at high levels and show no response to CDDO-Im treatment. This provides further evidence that the KEAP1-NRF2 system is inducible in Chicken, but not Neoaves, with the latter manifesting constitutively high Nrf2 transcriptional activity across multiple target-genes.

4. In Figure 5, when comparing all neoaves with basal aves, the presence of functional KEAP1 was not associated with increased lifespan and basal metabolic rates (p=0.25). It does not seem fair to separate the Passeriformes, which have the same KEAP1 mutation.

Response

We appreciate this constructive criticism. We have conducted a new, more sophisticated analysis that directly compares basal Aves and Neoaves (Fig. 5C-D; lines 372-414). In summary, we find that basal metabolic rates (BMR) differ significantly between Neoaves and basal Aves species with equivalent lifespans (<10 years; 10-19 years); Neoaves with low BMR reach higher lifespans than any basal Aves (>50 years); and Neoaves with BMR higher than that of any basal Aves still maintain equivalent lifespans, despite a nearly four-fold increase in MS-BMR.

Minor comments

1. In Figure 1E, NRF2 protein levels should be compared between no treatment and KEAP1 Cas9. The blots should be shown on same membrane.

Response

We agree with this suggestion and have conducted a new western blot experiment using freshly extracted cell lysates. The analysis is now presented in the revised Figure 1E.

2. In Figure 1F, are the KEAP1-null HEK293T cells used for the transfection experiments? If so, basal ARE-luciferase activity should be high even in absence of NRF2 transfection.

Response

We thank the reviewer for pointing this out. Basal ARE-luciferase activity is indeed higher in the KEAP1-null HEK293T cells relative to WT, even in the absence of NRF2 transfection. This is shown in Fig. S4, panel C (DMSO, vehicle). We have also drawn attention to this in the text where the KEAP1-null HEK293T cells are first presented (line 165).

3. Figure 2B is not cited in text.
Response

We have reviewed the manuscript and confirm the initial citation of Figure 2B on line 202.

Reviewer #2:

In this manuscript, the authors demonstrated that functional Keap1 is lost at the start of Neoaves evolution and several gene variations coevolved to compensate for the constitutive Nrf2 activation. Furthermore, they demonstrated that the functional loss of Keap1 leads to longer longevity in birds, implying the possible way for human longevity enhancement. This manuscript is well organized, well written and exciting. I have only minor critics.

Response

We thank the reviewer for the positive comments and for recognizing the merits of our study. We also appreciate your time and consideration reviewing our paper.

Minor points:
1. In Figure 3E; the authors should indicate what fluorescence was measured in the figure legend.

Response

We thank the reviewer for identifying this. We have now added the exact fluorescence excitation and emission wavelengths in the Figure 3 caption (lines 1126).

2. Line 244 and 303; ARE is an abbreviation of antioxidant response element. Therefore, to say "ARE element" is redundant.

Response

We have corrected this redundancy in the manuscript (now lines 246 and 309).

3. Figure 2C; The authors indicated in the figure that "★(star mark) IgG background=undetectable" and indicated the ★(star mark) above each bar except for those for NRF2. This means that IgG background is detected at the NRF2 locus. However, the authors did not describe how they treated it to calculate the results. Furthermore, the mark above the PRDX1 looks like "asterisk" rather than the star mark.

Response

We thank the reviewer for identifying these mistakes and have provided a revised Figure 2C. We have corrected the *PRDX1* mark to be consistent with the others. We have also thoroughly reviewed our source data for the graph in Figure 2C and have found that of the two NRF2 AREs shown for chicken, only one had detectable IgG background. We have therefore added a star mark above this second NRF2 ARE bar. Furthermore, this bar was incorrectly formatted and reflected an incorrect value. This has been revised to reflect the true experimental value. Lastly, the caption for Figure 2 has now been revised to describe the exact IgG background detected at the second NRF2 locus, which is only 0.6% of the signal obtained with the NRF2 antibody.

4. Figure 2A; "Fold change NRF2 RNA expression" should read "Fold change NRF2 mRNA expression".

Response

This has been corrected.

5. Throughout the manuscript; Abbreviations should be fully spelled out only at their appearances.

<u>Response</u>

This has been corrected

Reviewer #3:

General comments:
This is a thoroughly conducted study presenting exciting results of interest to a broad audience. The authors set out to study why birds seem to have superb abilities to counteract oxidative stress (and live comparably long lives) despite excess ROS production caused by their ability to fly and high metabolic rates. The authors target their studies on a main transcription factor (NRF2) in the major cellular pathway that regulates the antioxidant response to intracellular oxidative stress. They find an astonishing difference between Aves (the basal bird groups) and Neoaves (makes up 95% of all bird species). In Aves the NRF2 gene is strongly regulated by a functional KEAP1 gene only allowing the NRF2 to trigger intra-cellular antioxidant defense during stressful conditions. In contrast, in Neoaves the KEAP1 gene is non-functional and the NRF2 gene always expressed resulting in a constitutive intra-cellular antioxidant defense, and they also show that in Neoaves NRF2 proteins are located (constitutively!) in the cell nucleus! This could arguably provide Neoaves with super-efficient antioxidant abilities, which may have facilitated evolution of higher metabolic rates and possibly longer life span. The authors also show that there has been compensatory effects, for example a complete loss of a pro-tumerogenic locus in Neoaves (NQO1) that in Aves (chicken) is upregulated by NRF2, as well as loss of NRF2-binding regulatory sites upstream the GSTA2 gene (identified to be involved in bird song and plumage coloration pathways).
In conclusion, I really liked this study and how it throughout the Results section walked step-by-step to test (and compare between Aves and Neoaves) different aspects of the major antioxidant pathway where NRF2 is a key regulating factor. Super nice and interesting!

Still, I think there are some places where results have been over-interpreted, in particular in the Discussion (see my specific comments below), and the manuscript would become more solid with a careful revision on this point.

<u>Response</u>

We greatly appreciate Reviewer 3's enthusiasm regarding the potential importance of our study and at the same time acknowledge the valuable suggestions, including the importance of not over-stating our conclusions.

Specific comments:

1. Abstract: Here authors say: 'Our evidence suggests….' '…that this ancient mutation induced a compensatory program in NRF2-target genes associated with feather development and plumage coloration, while enabling significant increases to life span and metabolic rates.'
I agree that the authors have found some evidence for induced compensatory program in one feather development gene (β-keratin) that also may be involved in non-carotenoid plumage coloration. However, the authors talk about 'NRF2-target genes', so implying that they have been analyzing more than one gene. The other gene they investigated was the GSTA2 gene, which was the only gene they found being included in all three gene sets they obtained from the literature search (comparing genes involved in NRF2-regulation in mice, plumage coloration in Neoaves, zebra finch song system). However, I cannot imagine that research experts on bird 'plumage coloration' genes would say that GSTA2 is an important

gene in plumage coloration pathways. Most researchers interested in bird plumage coloration would think when they read this Abstract that the authors have investigated key genes involved in carotenoid- and melanin-based plumage colors. There is no such analysis. Revise this to avoid the impression that several genes, both feather development and plumage coloration genes, have been investigated in this study. I also have some problems with the ending of the sentence in the Abstract quoted above. I am not convinced that the constitutive NRF2 gene activity has had any effect on MS-BMR or maximal life span. The most rigorous test for the purpose of this study is to compare maximal life span between Aves (with KEAP1-regulated NRF2 gene expression) and Neoaves (with constitutive, non-KEAP1 regulated, NRF2 expression) and this test is not significant (p=0.25). And I am not convinced that the follow-up test really shows the claimed significant differences between Aves and Neoaves (see my worries below under Page 15, 3d part).

Response

We appreciate these very important criticisms. We agree that the previous abstract led to the impression that multiple melanin- and carotenoid-based genes were investigated. We have therefore replaced "plumage" with "feather development" in the title, to reflect the β-keratin analysis. We have also removed "plumage coloration" from the abstract and other appearances in the text (lines 23, lines 283-284). The reviewer's comment regarding our analysis of the possible effect of constitutive Nrf2 activity on MS-BMR and maximal life span is well taken and led us to perform additional analyses, which we describe in greater detail under comment #3 below. Accordingly, we have revised the abstract to reflect our new analyses on maximum lifespan and MS-BMR in basal Aves vs Neoaves. Briefly, this new analysis has identified significant increases in MS-BMR between Neoaves and Aves, with no significant trade-off cost on Neoavian lifespan.

2. Page 14, 2nd part, line 7: Here you say that '…these analyses provide in vivo evidence that the evolution of NRF2-associated feather and song system genes has been shaped by the constitutive activation of NRF2 in Neoaves.' However, the only data on any song system related gene is the GSTA2 gene – and as far as I know this is not a key gene in the song system pathway. I suggest you omit 'song system genes' from this sentence. It becomes too much of hand-waiving and over-interpretation of your data – in fact, it takes credit from your meticulous work on the KEAP1 and NRF2 in Aves vs. Neoaves!

Response

We really value this advice. We have removed "song system genes from this sentence (lines 353-354).

3. Page 15, 3d part, lines 2-9: For the most correct and rigorous test, comparing either life span or MS basal metabolic rate between functional KEAP1 (Aves) vs. non-functional KEAP1 (Neoaves) bird species, no correlations were found (p=0.25). The authors argue that there was a trend for Neoaves to have higher values in both tests, and this may be hinted when examining Figure 5A and 5B by eye. However, no means and SD are presented anywhere in the paper. Moreover, the statistics presentation in Table S11 is impossible to read and understand because it is not possible to connect each response variable (life span or MS-BMR) to the correct row of test information in the Table. The Table S11 needs to be revised for clarity! Then, the authors divide the KEAP1 factor into three groups and run MANOVA based on this grouping for the two response variables (still impossible to separate in the table). This yields an overall significant result. However, the problem with this is that we do not know if the significant difference is between Aves and the two Neoaves groups, or if the significant difference is within Neoaves (i.e., between Passerines and non-passerine Neoaves). Inspecting the figures by eye suggest to me that the difference is between Passerines and non-passerine Neoaves both for life span and MS-BMR. If so, this would result in a different conclusion than the one given in the present version of the MS. If you think that Passerines are so different in aspects of size etc., you may in the follow-up analysis exclude Passerines and just compare

Aves with non-passerine Neoaves. If in these analyses non-passerine Neoaves shows significantly higher life span and MS-BMR, then you have a case for that constitutive NRF2 activity may increase life span and MS-BMR in non-passerine Neoaves.

Response:

We thank the Reviewer for these highly constructive criticisms. We agree that the most correct and rigorous test would be comparing basal Aves and Neoaves. We have therefore conducted a new analysis that directly compares basal Aves (BA) and Neoaves (NA), with means and standard errors presented (Fig. 5C-D; lines 372-414). To increase our statistical power, we have stratified the MS-BMR and lifespan data. This allows us to conduct two complementary analyses. The first compares MS-BMR between BA and NA species of equivalent lifespan (Fig. 5C), and the second investigates lifespan between BA and NA species of equivalent MS-BMR (Fig. 5D). (Please note that the stratified data is non-normal, and therefore our new analysis employs the non-parametric Kruskal-Wallis test, again, within a phylogenetic framework utilizing simulations). The new results are summarized in the newly revised table S11. In summary, we find that:

(1) MS-BMR is significantly increased in Neoaves within two lifespan groupings (<10 years; 10-19 years)
(2) Neoaves with low MS-BMR can reach maximum lifespans beyond the range of any basal Aves, even when these basal Aves have equivalent MS-BMR.
(3) NA with MS-BMR higher than that of any BA still maintain equivalent lifespans, despite a nearly four-fold increase in MS-BMR.

This suggests that the constraints imposed on lifespan by MS-BMR (Fig.5E) have been relaxed in Neoaves, potentially explaining why significant increases to MS-BMR have had no discernable effect on lifespan during the Neoavian radiation. Since our analysis presents statistical evidence that the loss of KEAP1 was associated with significant increases to metabolic rates, this raises the intriguing hypothesis that constitutive NRF2 activity may have facilitated the simultaneous diversification of Neoavian MS-BMR and lifespan.

4. Page 16, 2nd part, lines 5-7: Here authors say: '…induced wide-ranging compensatory mutations in NRF2 target-genes associated with plumage coloration and feather development…'. I think it is an over-statement to talk about 'wide-ranging compensatory mutations' when only two genes have been analysed, and only one of these is clearly important in feather development (the gene argued to be involved in plumage coloration (GSTA2) is in fact a gene known to be importantly involved in ROS scavenging). Revise this to avoid over-statement.

Response:

We have revised this sentence to avoid over-statement. The sentence now mentions only "…induced compensatory mutations in NRF2 target-genes with functions beyond redox regulation—including feather development" (line 425-428)

5. Page 16, 2nd part lines 7-8: Here authors write: '…statistically significant increases in avian life span and metabolic rates.' I am not convinced by the statistical analyses used to test this (see my argumentation above under page 15, 3d part). Please reanalyse this.

Response:

We have revised this sentence to reflect our reanalyzed results (lines 426-428)

Page 18 1st part, lines 1-3: See previous point!

Response:

This has been corrected to reflect our reanalyzed results (lines 462).

Page 19, 2nd part, line8: Instead of citation 53, better cite the original idea paper by von Schantz et al. 1999 ('Good genes, oxidative stress and radical sexual signals' in Proceedings of the Royal Society, B 266:1-12).

Response:

We thank the reviewer for bringing this paper to our attention. We have replaced the original citation 53 with von Schantz et al. 1999 (lines 500; 944-946).

Reviewer #1 (Remarks to the Author):

The authors did not address most of main points from this reviewer. While this reviewer understand the difficulty to generate NRF2 knockout birds and to perform microarray analysis of bird genes, the reviewer would still like to recommend to conduct NRF2 siRNA knockdown experiments using Neoaves primary cells before publishing this paper.

Reviewer #2 (Remarks to the Author):

The authors have adequately responded to the reviewer's concerns. I have no further critics.

Reviewer #3 (Remarks to the Author):

In my view, the authors have made a thorough revision that has resulted in an even stronger and more convincing study. New lab analyses have been conducted, a considerable revision of the statistics have been done, and the interpretations in the Discussion have been revised now matching the actual results of the study in a better way. In particular, I think that the complete statistical reanalysis of the comparison of life span and basal metabolic rate between basal Aves and Neoaves provided much stronger support for the conclusion that Neoaves species can achieve longer life span with similar (or even higher basal metabolic rates).
I find that the authors have responded in a satisfactory way to all the points I raised in my review of the first version of this manuscript.
In conclusion, I congratulate the authors to a thoroughly executed study that provides very interesting data that surely will exciting to a broad range of researchers and inspire new research projects.

REVIEWERS' COMMENTS:

Reviewer #1 (Remarks to the Author):

The authors did not address most of main points from this reviewer. While this reviewer understand the difficulty to generate NRF2 knockout birds and to perform microarray analysis of bird genes, the reviewer would still like to recommend to conduct NRF2 siRNA knockdown experiments using Neoaves primary cells before publishing this paper.

Response

We appreciate the Reviewer's appreciation of the difficulty involved with these suggested experiments. Indeed, the siRNA knockdown experiments would be very useful in further establishing the role of NRF2 in regulating feather beta-keratin genes. However, it is unclear whether an *in vitro* cell culture analysis of feather beta-keratin gene expression would be biologically informative, since these genes and their regulation has traditionally been studied *in vivo* (e.g. Wu et al. 2015, PNAS, E6770–E6779). We believe that our study provides an important first step by identifying NRF2 occupancy of feather beta-keratin antioxidant response elements, *in vivo*, within skin samples of wild Neoaves individuals, and hope that our study will spur future extensive in vivo studies that further develop this line of investigation.

Reviewer #2 (Remarks to the Author):

The authors have adequately responded to the reviewer's concerns. I have no further critics.

Response

We thank the Reviewer for their valuable comments of previous drafts of the manuscript.

Reviewer #3 (Remarks to the Author):

In my view, the authors have made a thorough revision that has resulted in an even stronger and more convincing study. New lab analyses have been conducted, a considerable revision of the statistics have been done, and the interpretations in the Discussion have been revised now matching the actual results of the study in a better way. In particular, I think that the complete statistical reanalysis of the comparison of life span and basal metabolic rate between basal Aves and Neoaves provided much stronger support for the conclusion that Neoaves species can achieve longer life span with similar (or even higher basal metabolic rates).
I find that the authors have responded in a satisfactory way to all the points I raised in my review of the first version of this manuscript.
In conclusion, I congratulate the authors to a thoroughly executed study that provides very interesting data that surely will exciting to a broad range of researchers and inspire new research projects.

Response

We thank the Reviewer for the extremely helpful comments on the previous draft of the manuscript. We very much appreciate the encouragement and support.
